# Development of bacteria-based bioorganic phosphate fertilizer enriched with rock phosphate for sustainable wheat production

**DOI:** 10.3389/fmicb.2024.1361574

**Published:** 2024-07-23

**Authors:** Zoya Aslam, Mahreen Yahya, Hafiz Shahid Hussain, Saira Tabbasum, Sabahet Jalaluddin, Shazia Khaliq, Sumera Yasmin

**Affiliations:** ^1^Soil and Environmental Biotechnology Division, National Institute for Biotechnology and Genetic Engineering College, Pakistan Institute of Engineering and Applied Sciences (NIBGE-C, PIEAS), Faisalabad, Pakistan; ^2^Botany Department, Bahauddin Zakerya University, Multan, Pakistan; ^3^Industrial Biotechnology Division, National Institute for Biotechnology and Genetic Engineering College, Pakistan Institute of Engineering and Applied Sciences (NIBGE-C, PIEAS), Faisalabad, Pakistan

**Keywords:** BOP, microcosm, rock phosphate, plant material, heat-tolerant phosphate-solubilizing bacteria

## Abstract

Phosphorous (P) is a limiting macronutrient for crop growth. Its deficiency prevents plant development leading to an extensive use of phosphatic fertilizers globally. Bio-organic phosphate (BOP) fertilizer provides a sustainable approach to optimize nutrient availability, enhance crop yield, and mitigate the negative impacts of chemical fertilizers on the environment. Therefore, the present study integrates the application of heat-tolerant phosphate-solubilizing bacteria, rock phosphate, and organic materials for the development of BOP. For this purpose, potential heat-tolerant phosphate-solubilizing bacteria (PSB) were isolated from major wheat-growing areas of southern Punjab. Five isolates were the efficient phosphate solubilizers based on *in vitro* phosphate-solubilizing activity (291–454 μg ml^−1^ and 278–421 μg ml^−1^) with a concomitant decrease in pH (up to 4.5) at 45°C and 50°C, respectively. These PSB were used for the development of potential consortia that are compatible and showed high P solubilization. *In planta* evaluation of these PSB consortia in a pot experiment under net house conditions showed that consortium-2 had a favorable impact on growth parameter with enhanced grain yield (9.63 g plant^−1^) and soil available P (10 μg g^−1^) as compared with 80% uninoculated control. The microcosm study was conducted to evaluate PSB consortium-2 integrated with carrier material (plant material and filter mud) and rock phosphate as BOP increased total phosphorous (14%) as compared with uninoculated controls. Plant-based BOP showed higher viable count (3.5 × 10^8^\u00B0CFU) as compared with filter mud-based BOP. Furthermore, the effect of BOP on wheat growth parameters revealed that BOP showed a promising influence on grain yield (4.5 g plant^−1^) and soil available P (10.7 μg g^−1^) as compared with uninoculated 80 and 100% controls. Principle component analysis (PCA) further validates a positive correlation between BOP with grain weight and plant height and soil available P as compared with both 80 and 100% controls. For the first time, this study reports the combined application of bio-organic phosphate fertilizer and heat-tolerant PSB, which offers an eco-friendly option to harvest better wheat yield with low fertilizer input.

## Introduction

Phosphorous (P) is the second most essential macronutrient after nitrogen due to its direct and indispensable role in fostering plant growth and enhancing crop yield (Lynch, 2007; Lambers et al., 2011; Shen et al., 2011). It constitutes approximately 0.2 to 0.8% of plant dry weight (Sharma et al., 2013; Elhaissoufi et al., 2022) and is actively involved in essential plant physiological processes (Siedliska et al., 2021) and vital biological processes such as cell division, nucleic acid formation, and tissue growth (Vance et al., 2003). Soil available P exists in various organic and inorganic forms, but the available P was poor in the majority of the soils for plant uptake. Despite the fact that soil typically contains 0.05% of (w/w) P, merely 0.1% of this fraction is available for plant uptake (Zhu et al., 2011). Reduced P availability is primarily attributed to lower organic matter levels, high pH (high calcium content), and suboptimal fertilizer use efficiency (Tabbasum et al., 2021). In Pakistan, arid climatic conditions, minimal organic matter content, and alkaline calcareous nature of soils are crucial factors contributing to diminished nutrient availability and reduced yield. Therefore, limited P availability in agricultural soil is a significant obstacle in sustainable agricultural management.

Globally, more than 40% of soils are deficient in P. To address this issue, phosphorous is externally applied through chemical phosphate fertilizers (Aimen et al., 2022). However, only 20% of applied chemical P fertilizers is beneficial to plants (Plaxton and Tran, 2011; Ibrahim et al., 2022), while the rest of applied chemical P fertilizers either accumulates in soil as insoluble mineral complexes or is separated from the soil (Wakelin et al., 2004; Bouizgarne et al., 2023). The increased awareness about the negative impacts of chemical fertilizers on both human health and the environment has led to a growing interest in more sustainable eco-friendly agricultural practices. Hence, it is imperative to use approaches that have the potential to elevate the availability of P for uptake by plants from agricultural soils.

Rock phosphate (RP) is the primary source of P fertilizers (Kari et al., 2019). Consequently, it is noteworthy and beneficial to explore environment-friendly strategies that involve the direct application of rock phosphate (Azaroual et al., 2020; Bouizgarne, 2022). While rock phosphate is permissible for use in crop production, its effectiveness as a source of P is constrained. Reduced RP efficiency is attributed to elevated pH levels and low organic matter content (Richardson et al., 2011; Cicek et al., 2020). Therefore, in the fertilizer production process, RP is treated with strong acids, mainly sulfuric acid, to solubilize P (United, S, and Tennessee Valley, 1964; de Oliveira Mendes et al., 2020), whereas this process is expensive and causes environmental pollution (Xiao et al., 2019).

Phosphate-solubilizing bacteria (PSB) are a promising alternative to solubilize RP (Goldstein et al., 1993; Vassilev et al., 2014; De Oliveira Mendes et al., 2020). These bacteria convert insoluble phosphates into plant-available forms by producing organic acid, engaging in cation chelation or participating in exchange reactions (Chung et al., 2005; Gulati et al., 2010; Tian et al., 2021). Most PSB exhibit other characteristics of plant growth-promoting bacteria such as nitrogen fixation, zinc solubilization, indole acetic acid (IAA) or phytohormone production, and aminocyclopropane-1-carboxylic acid (ACC) deaminase activity (Wei et al., 2018; Timofeeva et al., 2022). Furthermore, the addition of organic material modifies soil porosity, soil pH, water holding capacity, and microbial activities, and this phenomenon increases P availability in plants (Wang et al., 2016). Enhanced mobility of phosphorus in soils amended with organic matter and PSB is attributed to stimulated microbial activity, release of organic acids, and mineralization of organic matter.

RP combined with PSB can serve as a P source. However, the bacterial P solubilization efficiency can be enhanced by supplementing with organic manure or organic compost (Ditta et al., 2018). Additionally, the organic matter improves soil physicochemical properties, increases nutrient levels, and improves soil quality. Therefore, the integrated application of RP, PSB, and organic material is recommended as a promising approach for sustainable plant growth and production. Bio-organic fertilizer derives from organic materials and contains living microorganisms, such as bacteria, fungi, and other beneficial organisms that enhance soil fertility and promote plant growth. Gao et al. (2020) reported a significant increase in growth, yield, and nutrient uptake of maize (*Zea mays* L.) crop as a result of the application of bio-organic fertilizer comprised of *Azotobacter chrocoocum*, arbuscular mycorrhizal fungi (AMF), and *Bacillus circulans* along with organic materials, such as biogas slurry and humic acid. Previous studies demonstrated that the combined application of compost, biogas, RP, and *Bacillus* strain led to enhanced yield of bread wheat with low fertilizer input under arid climatic conditions (Tahir et al., 2018). Another study reported that wheat growth parameters can be enhanced with increased grain P content by using compost enriched with RP and *Bacillus thuringiensis* (Khakwani et al., 2017). However, to the best of our knowledge, limited information is available about the effect of the combined application of RP, organic materials, and consortium of thermotolerant PSB on soil fertility and the growth and productivity of contrasting wheat cultivars grown at different fertilizer levels.

Therefore, the current study focuses on integrating the application of heat-tolerant phosphate-solubilizing bacteria, RP, and organic materials for the development of bio-organic phosphate (BOP) fertilizer under natural conditions. We hypothesized that (H1) co-composting of plant material with RP and PSB enhances nutrient (P) availability. We assumed (H2) that BOP increases grain yield in wheat and enhances available P in soil with reduced application of chemical fertilizer.

## Materials and methods

### Sample collection

Rhizosphere soil samples of field-grown wheat were collected from different districts of southern Punjab, i.e., Bahawalnagar (29.348194639076453, 72.9951854271594), Multan (30.268453, 71.415518), Muzaffargarh (30.282401, 71.391901), Sialkot (32.42979952823552, 74.60978258215574), Jhang (31.320934290652488, 72.59942827525828), and Hyderabad (31.346443525303936, 71.6827859886904). For the isolation of PSB, soil samples were transported to the laboratory and stored at 4°C until further processing.

### Isolation of heat-tolerant phosphate-solubilizing bacteria

For the isolation of heat-tolerant PSB, soil samples were enriched in National Botanical Research Institute’s Phosphate broth medium [NBRIP; glucose 10 g, magnesium chloride 5 g, tri-calcium phosphate (TCP) 5 g, potassium chloride 0.20 g, (NH_4_)_2_SO_4_ 0.10 g, magnesium sulfate (MgSO4.7H_2_O) 0.25 g, agar 15 g, and distilled water 1 L, and the pH was maintained to 7.0 ± 0.2] at different temperatures (45° C to 60° C). These suspensions were then vortexed and serially diluted from 10^−2^ to 10^−4^. Aliquots of each dilution were spread on LB agar plates. These plates were kept in an incubator at 30 ± 2°C for 24–48 h. To get pure colonies or isolates, morphologically distinct colonies were selected and sub-cultured after incubation. For the calculation of colony forming unit (CFU), the formula described by Somasegaran and Hoben (2012) was used. Cell and colony morphology, gram staining, and motility of bacterial isolates were analyzed using stereo microscopy and light microscopy (Vos et al., 2011).

Blood agar medium was used for the biosafety assessment of selected PSB. The formation of clear zone around the bacterial cultures indicates the hemolytic activity of the isolates (Russell et al., 2006). Blood agar plates with 5% v/v sheep blood (commercially available) were used for this purpose. Blood agar plates were streaked with PSB fresh cultures and stored in an incubator at 37 ± 2°C for 48 h. The absence of zone formation around bacterial colonies indicates the non-hemolytic activity of isolates and they are safer to use.

### Screening of inorganic phosphate solubilization

To evaluate the phosphate solubilizing ability of isolates, pure isolated bacterial cultures were grown in LB broth medium (10 μL; 10 g tryptone, 5 g NaCl, 5 g yeast extract, 20 g agar, 1 L distilled water, and pH maintained to 7 ± 0.2) for 24 h and spotted on NBRIP agar medium (Senthilkumar et al., 2021) comprising of tricalcium phosphate (TCP) as inorganic phosphate source. A loopful of these pure bacterial colonies was picked from the LB agar plates and spotted on the NBRIP agar plates. These plates were incubated at 30 ± 2°C in an incubator for 6 to 7 days, and the development of halo zone surrounding the spotted bacterial colonies was observed. The solubilization index was calculated by using the following formula described by Paul and Sinha (2017).

### Quantitative estimation of phosphate solubilizing activity

For the quantification of phosphate solubilizing activity of bacteria, the molybdate blue method was used (Suleman et al., 2018; Yahya et al., 2021). Bacterial cultures were cultivated in NBRIP broth medium at 45° C and 50°C and placed on a shaker at 150 rpm for 7 days. The cell-free supernatant was obtained by centrifuging cultures at 4°C for 10 min at 4,000 rpm. The optical density (OD) was recorded using a spectrophotometer (Camspec M-350 double beam UV visible spectrophotometer, United Kingdom) at 882 nm. Phosphate solubilization was determined using a KH_2_PO_4_ standard curve with standards of 2, 4, 6, 8, 10, 15, 20, 50, and 100 ppm. To quantify phosphate solubilization, the optical density (OD) was plotted on a graph against the standard solution concentration (μg ml – ^1^; Soltanpour and Workman, 1979; Vos et al., 2011). A pH meter (Pvhs-3c, Rex, China) was used to assess the pH of the cell-free supernatant.

### Molecular identification of isolates based on 16S rRNA gene

PSB were identified by amplification and sequencing of 16S rRNA gene. Selected PSB strains were streaked on LB agar plates and placed in an incubator at 30 ± 2°C. The LB broth was inoculated with single pure bacterial colony and placed on a shaker overnight (140 rpm) at 30 ± 2°C. After incubation, pellet was collected by centrifuging 1 mL of bacterial culture for 10 min at 13,000 rpm. The CTAB method was used to isolate DNA from the pellet (William et al., 2012). 16S rRNA gene of isolates was amplified using PCR with a reaction mixture containing 5 μL of Green PCR Master Mix (Thermo Scientific), 2 μL of template DNA, 2 μL of PCR water, 0.5 μL of reverse primer rD1 (5’ AAGGAGGTGATCCAGCC 3′), and 0.5 μL of forward primer fD1 (5’ AGACTTTGATCCTGCTCAG 3′). The PCR profile was optimized as follows: initial denaturation for 5 min at 95°C followed by 30 cycles of denaturation for 60 s at 95°C, annealing for 1 min at 53°C, extension for 60 s at 72°C, and then final extension for 10 min at 72°C. The amplified PCR product was then run on 1% agarose gel in 1X TAE buffer containing 20 mg ml − ^1^ of ethidium bromide. The gel was run on an electrophoresis apparatus at 80 V for 30 min. A ladder of 1 kilo base pairs (1Kb) was used as a size marker. Then, a gel documentation system (Vilbour Lourmat CN 1000 26 MX) was used for gel visualization. The PCR product was purified using QIAquick kit (QIAGEN Sciences, Maryland 20874, United States). The purified PCR product was then sequenced commercially in Punjab Institute of Nuclear Medicine (PINUM), Faisalabad, Pakistan. The acquired sequences were compared with type strains using the Basic Local Alignment Search Tool (BLAST). The results of percentage identity, homology, and E-values were recorded. Following that, the sequences were submitted to the National Center for Biotechnology Information (NCBI), GenBank,^1^ and the accession numbers were obtained.

### Bioassay for the detection of plant growth-promoting activities of bacterial isolates

Aminocyclopropane-1-carboxylic acid (ACC) deaminase activity of PSB strains was assessed in Dworkin and Foster (DF) salt minimal medium (Penrose and Glick, 2003) containing 30 μL of 0.5 M ACC as a sole nitrogen source. Minimal salt solution comprising of zinc salt (ZnO) was used for assessing the ability of PSB to solubilize zinc. The formation of halo zone around PSB colonies indicated zinc solubilization by the bacterial isolates. The solubilization index was calculated using the formula described by Paul and Sinha (2017).

The colorimetric method was used for detecting the IAA production by isolates (Lasudee et al., 2018). For this purpose, bacterial colonies were cultivated overnight in an LB broth supplemented with tryptophan (0.01%). The broth was then placed in an orbital shaker at 110 rpm for 7 days. IAA was produced using Salkowski’s reagent. The development of pink color indicates the production of IAA by the isolates (Gordon and Weber, 1951). The nitrogen fixing ability of the bacterial isolates was determined by observing the growth on a semi-solid N-free medium (Hardy et al., 1968).

To determine the production of exopolysaccharide (EPS), the selected isolates inoculated in the De Man–Rogosa–Sharpe (MRS) medium supplemented with sucrose (20% w/v) were kept in a shaking incubator at 37 ± 2°C for 48 h. A small amount of culture was run on a thin layer chromatography (TLC) plate (Silica gel 60 F254; Merck, Darmstadt, Germany) for 6 h, after which it was air dried, and the sugar spots were visualized by adding a solution containing 5% sulfuric acid in methanol (Roset et al., 2006).

### Development of PSB consortia

For the development of consortia, initially all the selected strains along with well-characterized strains were tested for the *in vitro* compatibility test (Irabor and Mmbaga, 2017). The bacterial inoculum was spotted on an LB agar plate and spread with the tested strain. The plates were incubated at 28°C ± 2°C for 72 h. No inhibition zone was observed around bacterial colonies, which indicate that strains are compatible to each other.

Three different consortia were prepared using some efficient strains from NIBGE Biotech Resource Center (NBRC) such as *Streptomyces* sp. R1 (Ashraf et al., 2022), BR123 (Ashraf et al., 2020) (GenBank accession numbers OL744553 and MT799988), *Brucella* sp. CH4 (GenBank accession number OR936580), *Bacillus* sp. A2, and *Ochrobactrum* sp. SSR (Rasul et al., 2021) (GenBank accession number MK422612). Consortium-1 comprised of *Pantoea cypripedii* ZR1, *Lysinibacillus fusiformis* ZR2, *Bacillus* sp. A2, and *Streptomyces* sp. R1 and BR123. Consortium-2 comprised of *Pantoea* sp. ZR1, *L. fusiformis* ZR2, *Enterobacter* sp. ZR3, *Klebsiella* sp. ZR4, and *B. haematophilia* CH4. Consortium-3 comprised of *P. cypripedii* ZR1, *Enterobacter quasihormaechei* ZR3, *Ochrobactrum* sp. SSR, *Bacillus* sp. A2, and *Streptomyces* sp. R1. Single colony of each bacterium was grown separately in an LB medium at 28 ± 2°C for 24 h. Equal volume of each bacterium grown in LB medium was mixed to develop the consortium (1 × 10^9^ CFU ml − ^1^).

### *In planta* evaluation of PSB consortia for P uptake and wheat growth

The effect of PSB in consortium-1, consortium-2, and consortium-3 on the recommended wheat variety, Gandum-7, was assessed in a pot experiment under net house conditions at NIBGE, Faisalabad (31°25′0″N 73°5′28″E), in the wheat growing season from November 2021 to April 2022.

Seeds were surface-sterilized and rinsed thrice with autoclaved dH_2_O. Sterilized seeds were soaked in consortium-1, consortium-2, and consortium-3 suspensions separately with 1 × 10^9^\u00B0CFU ml − ^1^ for 30 min. Uninoculated seeds soaked in a sterilized LB broth were used as a control.

Before seeding, all of the pots were watered to ensure the proper germination of seeds. Seeds were sown in pots (30 cm in diameter with 6 seeds per pot). The experiment was arranged in a completely random design (CRD) and consisted of three treatments, each with three replicates. The recommended doses of fertilizers comprising of nitrogen (150 kg ha^−1^) in the form of urea and P (100 kg ha^−1^) in the form of DAP were applied to the soil. Overall, 80% of the recommended DAP dose (20% reduced than the recommended dose) was added to treatments that included bacterial inoculation. As a control, pots with no inoculation were treated with 100% DAP and 80% DAP. Every treatment received 100% urea as a supplement. Plants were uprooted 65 days after planting and evaluated for growth parameters. The plants were harvested in April 2023 to examine growth, yield, and plant P content. The soil used for *in planta* evaluation was collected and homogenized properly for measuring organic matter, pH, P content, exchangeable potassium, and electrical conductivity (Ramanaiah et al., 2021). The total phosphorous content was determined by the method described by Andersen (1976).

### Microcosm studies for solubilization of RP in BOP formulations

For the development of different formulations of BOP, the efficient PSB consortium (consortium-2) was selected from *in vivo* studies, and a microcosm study was conducted. Different sources of organic matter, i.e., filter mud and plant material and RP as a substrate in 60:40 ratio were used to prepare two different types of BOP formulations. For the formulation of BOP, 46.8 g of plant material or filter mud and 31.2 g of RP were added to reagent bottles. Consortium-2 (22 mL) in 7:3 (w/v) ratio was inoculated in these reagent bottles. LB broth (22 mL) was added to bottles as a control. To maintain the moisture content, 30 mL and 60 mL distilled water were added to bottles containing filter mud and plant material, respectively. Two levels of moisture contents were maintained in both BOP formulations after 60 days of inoculation. There were eight treatments with three replicates (Supplementary Figure 3). The experiment was arranged in CRD. The bottles were then placed in an incubator at 37 ± 2°C. The viable count was conducted on 0, 7, 14, 30, and 60 days post inoculation (DPI), to ensure the survival of inoculated strains.

### Analysis of total P content of BOP formulation

For the analysis of total P (P_2_O_5_) in BOP formulations, 0.5 g of BOP fertilizer (BOP) sample was added to a volumetric flask (100 mL). Concentrated nitric acid (5 mL) and distilled water (50 mL) were added to the flask and set on a hot plate. Initially, the temperature of the hot plate was raised to 65°C for 30 min and then placed on a shaker for 60 min. After shaking, distilled water was added to get the required volume. The solution was filtered, and 10 mL of the filtrate was transferred to a conical flask; 5 mL of concentrated nitric acid and 15 mL (50%) of ammonium nitrate solution (50%) were added slowly while heating the contents on a hot plate at 65°C. Then, 50 mL of ammonium molybdate solution (3%) was added gradually while shaking vigorously overnight at room temperature. The precipitates were then filtered through a filter paper and rinsed with distilled water (ice cold). Washing was performed until the filtrate did not turn into blue litmus red (indicating that precipitates were not acidic). The precipitates were then thoroughly dissolved by adding 0.1 N NaOH (10 mL). Then, 2–3 drops of indicator (phenolphthalein) were added, resulting in the development of pink color. The mixture was titrated against 0.1 N H_2_SO_4_ with continuous shaking until a colorless mixture was obtained (Pakistan Standards and Quality Control Authority, 2021).

### Development of BOP under natural environmental conditions

**Figure 1:**
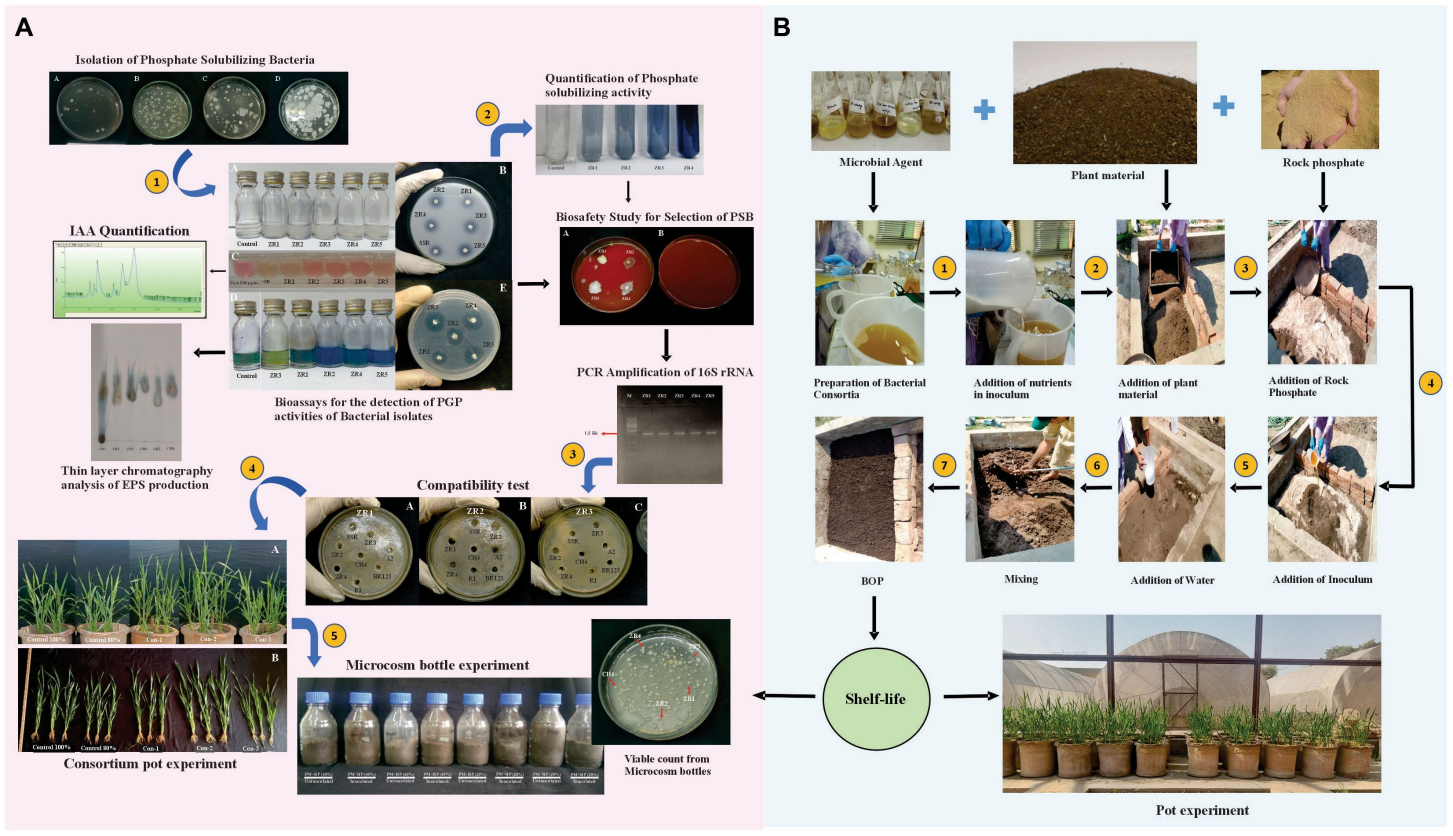
Illustration of the workflow of isolation and characterization of phosphate-solubilizing bacteria, compatibility test, pot experiment, and microcosm study **(A)** and development of BOP under natural environmental conditions **(B)**.

Most efficient BOP formulation was further developed and evaluated under natural environmental conditions in microplots at NIBGE, Faisalabad (31°25′0″N 73°5′28″E; Figure 1). Selected bacterial strains were inoculated in LB broth medium and placed oversight on a shaker at 180 rpm. *Streptomyces* spp. R1 and BR123 were cultivated in a starch casein broth at 30°C and shaken at 180 rpm for 7 days.

Initially, 30 kg of plant material was the first layer followed by a second layer of 20 kg RP in the microplot. Glucose (100 g), casein (30 g), and starch (50 g) were added onto the layers. After the addition of 6.5 L inoculum and 33 L water (to maintain 20% moisture content), the BOP was mixed thoroughly. The BOP was mixed every 7th day, and the samples were collected on 0, 7, 14, 30, and 60 DPI to study the shelf-life. Total nitrogen, phosphorous, potassium, pH, moisture content, and EC of BOP formulation were recorded at 60 DPI.

### Evaluation of BOP for wheat yield parameters

BOP formulation was further evaluated on the recommended wheat variety, AKBAR-2019, in a pot experiment under net house conditions at NIBGE, Faisalabad (31°25′0″N 73°5′28″E) in the wheat-cultivating season from November 2022 to April 2023. Seeds were surface-sterilized and then rinsed thrice with autoclaved dH_2_O. The plant-based BOP was applied to the topsoil of pots for soil amendment.

Before seeding, all the plants of the pots were watered to ensure the proper germination of seed. Seeds were sown in pots (30 cm in diameter; soil: sandy loam; pH 7.5). Each pot consisted of six plants. The CRD experiment consisted of three treatments, each with three replicates. The recommended doses of fertilizers were applied (N: P, 150: 100 kg ha^−1^). Overall, 80% of the recommended DAP dose was added to treatments that included BOP. As a control, pots with no amendment were treated with both 80 and 100% DAP. Every treatment received a dosage of 100% urea. The plants were harvested on April 2023, to examine growth, yield, and P uptake by the plants.

### Detection of inoculated bacteria in soil

The inoculated bacterial population of rhizosphere wheat soil was observed using the viable count (Somasegaran and Hoben, 2012) and BOX-PCR (Basheer et al., 2016). The strains were identified using their distinct morphological characteristics and light microscopy. Re-isolated PSB were identified by comparing the morphological characteristic of inoculated bacteria with other plant growth-promoting attributes such as P solubilization, IAA production, and zinc solubilization (Yasmin et al., 2016). Strain-specific finger prints of re-isolated PSB were compared with that of pure colonies using BOX-A1R primer 5′ CTACGGCAAGGCGACGCTGACG 3′ (Basheer et al., 2016).

### Statistical analysis

Analysis of variance (ANOVA) was used to statistically analyze data from laboratory scale and pot experiments using STATIX software 10.0 (Tallahassee, FL, United States). The least significant difference (LSD) test was used to evaluate the differences between various treatments from laboratory scale and pot experiments at confidence levels of 1 and 5%, respectively. Principal component analysis (PCA) was used on various plant parameters using PAST software version 3.26.

## Results

### Isolation and screening of heat-tolerant PSB

Rhizosphere soils of wheat were collected from several regions of South Punjab for the isolation of heat-tolerant PSB. These soil samples were tested for physiochemical properties (Supplementary Table S1). The bacterial population was determined by calculating the viable count from each soil sample. Seventy-nine morphologically different colonies were isolated, and out of these, 21 colonies showed phosphate-solubilizing activity on NBRIP agar medium. The maximum population of PSB (12) was isolated by second NBRIP enrichment at 45°C, and no PSB was found in wheat rhizosphere soils from second NBRIP enrichment at 60° C. The screening of selected bacteria for P solubilization was performed using NBRIP agar medium. The formation of halo zone around bacterial colonies varied from 1.6 mm to 3.7 mm. Six isolates, i.e., ZR1, ZR2, ZR3, ZR4, ZR5, and ZR6 showed significant phosphate solubilization activity. The maximum phosphate-solubilizing activity was observed by isolates ZR4 and ZR5 (SI: 3.75 mm), followed by ZR3 (SI: 3.2 mm) and ZR2 (SI: 3.00). The least P solubilizing activity was observed in ZR1 (SI: 2.00 mm) and ZR6 (SI: 1.6; Supplementary Figure S1).

### Quantification of P solubilizing activity

**Figure 2:**
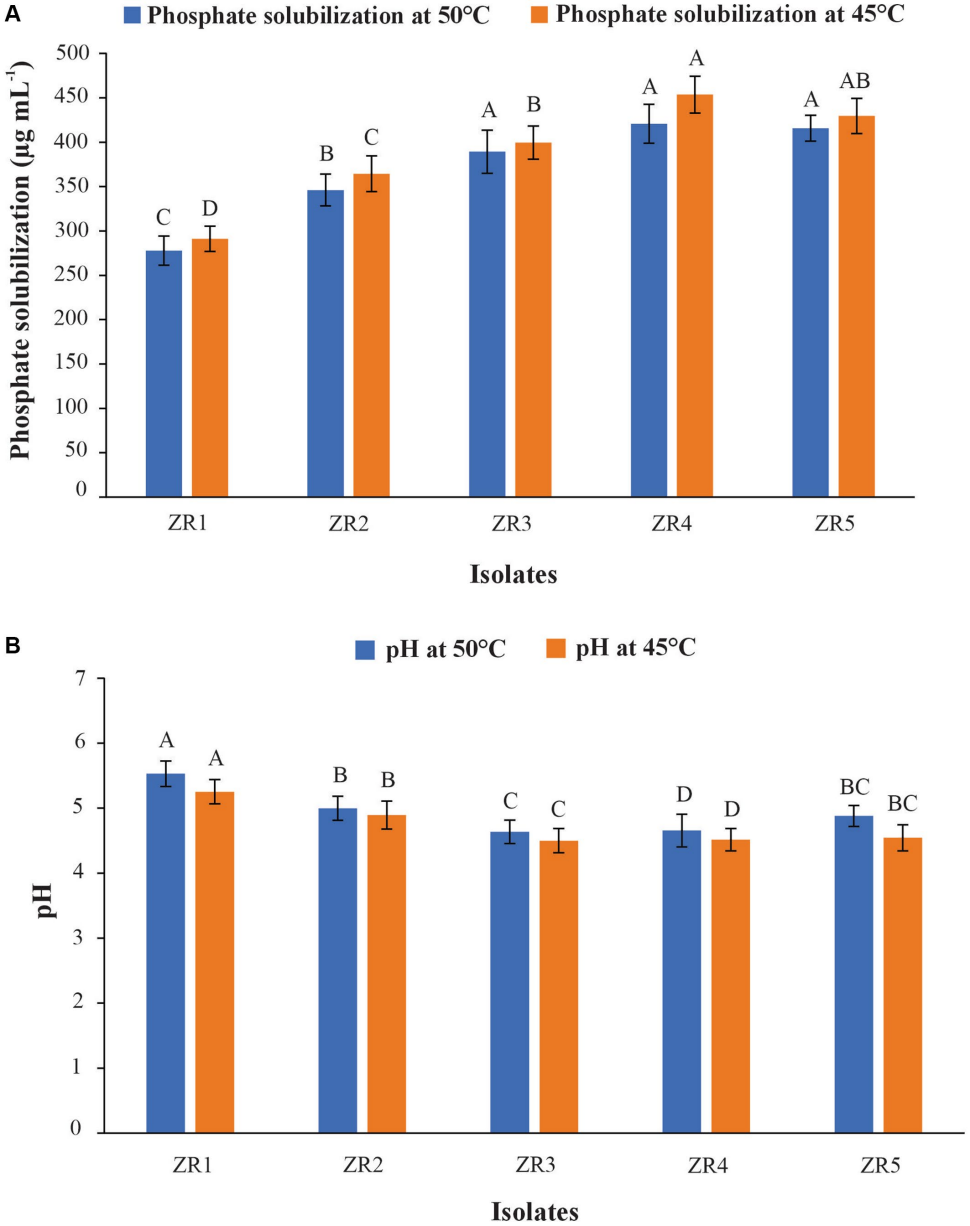
Phosphate solubilization **(A)** and pH changes **(B)** at 7 DPI by wheat rhizospheric bacteria in NBRIP broth added with heat-tolerant TCP at 45° C and 50° C.

In the quantification test, the maximum P solubilization activity (454 μg ml − ^1^) was conducted by isolate ZR4 followed by ZR5 (430 μg ml − ^1^) and ZR3 (400 μg ml − ^1^). The least amount of P (291 μg ml − ^1^) was observed in ZR1 (Figure 1). A decrease in pH of the medium was observed with the inoculation of different PSB. The maximum reduction in pH was observed in a filtrate of ZR4 (initial pH at 7 to 4.51) followed by ZR5 (initial pH at 7 to 4.54). A minimum decrease in pH was observed in ZR1 (initial pH at 7 to 5.25) at 45°C (Figure 2).

### Morphological and molecular characterization of PSB

All selected PSB (ZR1, ZR2, ZR3, ZR4, ZR5, and ZR6) were grown on Luria–Bertani (LB) agar medium. Some of the isolates, i.e., ZR1 formed circular colonies with a smooth surface. On the other hand, ZR3 colonies were off-white irregularly shaped with a flat surface. ZR2 formed a shiny white circular with convex elevation. ZR4 formed creamy white circular colonies with a raised surface. Pigmentation was not observed for all the tested strains. ZR1 was slow moving rod. ZR2, ZR4, and ZR5 were rod-shaped and non-motile. Cells of bacterial strain ZR3 were rod-shaped and showed fast movement with a slight whip-like movement under a microscope. Gram staining and light microscopy showed that all the tested strains (ZR1, ZR2, ZR3, ZR4, and ZR5) were gram-negative (Supplementary Table S2). Strains were identified as *Pantoea* sp., *Lysinibacillus* sp., *Klebsiella* sp., and *Enterobacter* sp. 16S rRNA gene sequences of *Pantoea* sp. ZR1, *Lysinibacillus* sp. ZR2, *Enterobacter* sp. ZR3, and *Klebsiella* sp. ZR4 and ZR5 were allocated with NCBI accession numbers of OR272290, OR272301, OR272303, OR272305, and OR272306, respectively (Supplementary Table S2). These PSB strains were submitted to NIBGE NBRC, Pakistan.

### Bioassays for the detection of PGP activities of bacterial isolates

PSB strains of *P. cypripedii* ZR1, *L. fusiformis* ZR2, *E. quasihormaechei* ZR3, and *Klebsiella* sp. ZR4 and ZR5 produced IAA as indicated by pink coloration in the reaction with Salkowski’s reagent (Supplementary Figure S1C). The qualitative spot test was carried out on ZR1 and ZR5 to produce light pink color. ZR2, ZR3, and ZR4 produced dark pink color (Table 1).

**Table 1:** PSB characterization for plant growth-promoting traits.

Sr.no	Phosphate solubilizing bacteria	Phosphate solubilized^1^	Indole acetic acid^2^	Growth on NFM^3^	Zn solubilization^4^	ACC deaminase^5^
		μg mL^−1^	Spot test	+/−	SI	+/−
1	*Pantoea cypripedii* ZR1	296 ± 15	+	+	2.8	+
2	*Lysinibacillus fusiformis* ZR2	374 ± 18	+++	+	6.2	+
3	*Enterobacter quasihormaechei* ZR3	403 ± 20	+++	-	8.75	+
4	*Klebsiella* sp. ZR4	457 ± 23	+++	+	8.25	+
5	*Klebsiella* sp. ZR5	437 ± 22	+	+	8.25	+

^1^P solubilization was detected quantitatively by the molybdenum blue method. ^2^IAA production was indicated by pink coloration upon reaction with Salkowski’s reagent. ^3^Nitrogen fixation ability was assessed by the growth of bacterial isolates in NFM. ^4^Zinc solubilization was confirmed by the formation of halo zone on an agar plate added with ZnO. ^5^ACC deaminase activity indicated by the growth of strains in vials supplemented with 0.5 M ACC.

Zinc-solubilizing activity was shown by *P. cypripedii* ZR1, *L. fusiformis* ZR2, *E. quasihormaechei* ZR3, *K. pneumoniae* ZR4, and *K. quasivariicola* ZR5 as indicated by the formation of halo zone around bacterial colonies on Tris minimal salt medium (Supplementary Figure S1E). The solubilization index (SI) ranged from 2.8 to 8.75 (Table 1). The maximum SI (8.75) was shown by ZR3 and the minimum SI (2.8) was observed for ZR1.

The nitrogenase activity was determined by changing the color of nitrogen-free medium (NFM) from green to blue in glass vials inoculated with *P. cypripedii* ZR1, *L. fusiformis* ZR2, and *Klebsiella* sp. ZR4 and ZR5 (Supplementary Figure S1D). *P. cypripedii* ZR1, *L. fusiformis* ZR2, *E. quasihormaechei* ZR3, and *Klebsiella* sp. ZR4 showed ACC deaminase activity, as indicated by the growth of strains in vials supplemented with 0.5 M ACC (Supplementary Figure S1A). Thin layer chromatographic analysis revealed that the isolate *P. cypripedii* ZR1 produced EPS using sucrose as a substrate (Figure 1).

### Biosafety study for the selection of PSB

A blood agar test was used to evaluate microorganisms for biosafety. When compared with the control, no blood lysis or formation of halo zone was detected in any of the selected PSB strains, showing that the bacteria are not harmful to humans and thus safe for further research (Supplementary Figure S2).

#### *In planta* evaluation of PSB consortia for P uptake and wheat growth

The evaluation of PSB in pot experiment at NIBGE in Faisalabad revealed that the PSB consortium-2 increased wheat plant fresh weight, shoot length, and root length (Table 2). Maximum yield was observed in pots inoculated with the consortium-2, i.e., *Pantoea* sp. ZR1*, L. fusiformis* ZR2*, Enterobacter* sp. ZR3*, K. pneumoniae* sp. ZR4, *and B. haematophilia* sp. CH4. These PSB increased grain yield (9.63 g plant^−1^) in comparison to the uninoculated 80% control. Soil available P of the consortium-2-inoculated treatment was significantly higher (10 μg g^−1^) as compared with uninoculated 80% control during harvesting (Table 3). The consortium-2-inoculated treatment significantly improved plant height and number of tillers as compared with both 80 and 100% uninoculated controls (Table 2).

**Table 2:** Effect of bacterial inoculation on plant growth parameters at different growth stages and wheat yield in the pot experiment under net house conditions.

At 35 DAS					At 65 DAS				At harvest stage					
Treatments	Root Length (cm)	Shoot Length (cm)	Fresh Weight (g)	Dry Weight (g)	Root Length (cm)	Shoot Length (cm)	Fresh Weight (g)	Dry Weight (g)	Plant height (cm)	Tillers	100 Grain weight (g)	Plant Biomass (g)	Grain yield (g / plant)	Viable count
Control 100%	5.53 ± 1.63 AB	20.73 ± 0.64 B	0.61 ± 0.04 B	0.11 ± 0.01 AB	14.33 ± 0.51 B	65.00 ± 1.11 B	20.03 ± 0.47 B	6.07 ± 0.15 B	75.33 ± 2.00 B	5.22 ± 0.44 A	4.57 ± 0.25 B	16 ± 0.66 B	8.5 ± 0.40 B	3.45 × 10^7^
Control 80%	3.40 ± 0.30 C	15.40 ± 0.26 E	0.35 ± 0.04 D	0.07 ± 0.02 C	9.93 ± 0.15 D	39.90 ± 1.15 E	14.40 ± 0.44 D	3.23 ± 0.06 E	66.22 ± 2.54 D	3.11 ± 0.33 C	3.36 ± 0.17 E	12.52 ± 0.59 D	5.37 ± 0.31 D	1.41 × 10^7^
Con-1	4.53 ± 0.40 BC	18.10 ± 0.10 C	0.50 ± 0.02 C	0.10 ± 0.01 ABC	12.57 ± 0.95 C	49.90 ± 0.17 C	16.63 ± 0.32 C	4.43 ± 0.31 C	72.33 ± 1.87 C	3.89 ± 0.33 B	4.32 ± 0.19 C	14.87 ± 0.80 BC	6.43 ± 0.45 C	3.32 × 10^7^
Con-2	6.13 ± 0.83 A	22.63 ± 0.25 A	0.93 ± 0.05 A	0.12 ± 0.03 A	16.30 ± 0.72 A	68.87 ± 0.15 A	25.43 ± 0.60 A	6.80 ± 0.10 A	79.00 ± 1.73 A	5.56 ± 0.53 A	4.83 ± 0.10 A	17.20 ± 0.80 A	9.63 ± 0.47 A	4.31 × 10^7^
Con-3	4.50 ± 0.10 BC	17.23 ± 0.21 D	0.57 ± 0.04 B	0.09 ± 0.01 BC	11.23 ± 1.01 CD	45.60 ± 1.55 D	13.93 ± 0.15 D	3.80 ± 0.10 D	64.22 ± 3.19 D	3.78 ± 0.44 BC	3.58 ± 0.32 D	13.70 ± 0.75 CD	5.83 ± 0.31 CD	1.73 × 10^7^

Effect of bacterial inoculation on various wheat growth parameters at 35 DAS and 65 DAS and during harvesting stage in a pot experiment at Faisalabad. Values represented are an average of three replicates. ± indicates standard deviation (SD). Means with significant differences (*p* < 0.05) among treatments are represented by different letters. Con-1: *Lysinibacillus fusiformis* ZR2, *Brucella haematophilia* sp. CH4, *Bacillus* sp. A2, *Pantoea cypripedii* ZR1, *Streptomyces* sp. strain R1, *Streptomyces* sp. strain BR123, and *Ochrobactrum* sp. SSR+ 80% DAP. Con-2: *Lysinibacillus fusiformis* ZR2, *Streptomyces* sp. BR123, *Streptomyces* sp. strain R1, *Brucella haematophilia* sp. CH4, *Bacillus* sp. A2, *Ochrobactrum* sp. SSR. + 80% DAP. Con-3: *Ochrobactrum* sp. SSR, *Bacillus* sp. A2, *Pantoea cypripedii* ZR1, *Enterobacter quasihormaechei* ZR3, *Streptomyces* sp. strain R1 + 80% DAP.

**Table 3:** Effect of bacterial inoculation on soil and plant P at different growth stages in the pot experiment under net house conditions.

	Soil available P (ppm)			Plant P (%)	
Treatments	At 35 DAS	At 65 DAS	At harvest stage	At 35 DAS	At 65 DAS
Control 100%	12.18 ± 0.12 B	9.24 ± 0.67 B	8.64 ± 0.06 B	3.83 ± 0.16 B	3.20 ± 0.10 A
Control 80%	9.73 ± 0.24 D	6.93 ± 0.29 D	6.01 ± 0.04 E	2.95 ± 0.40 D	2.31 ± 0.18 D
Con-1	10.49 ± 0.41 C	7.43 ± 0.42 CD	6.82 ± 0.37 C	3.17 ± 0.10 CD	2.75 ± 0.08 C
Con-2	13.88 ± 0.36 A	11.00 ± 0.65 A	10.05 ± 0.19 A	4.29 ± 0.23 A	3.00 ± 0.08 AB
Con-3	10.28 ± 0.52 CD	8.04 ± 0.71 C	6.45 ± 0.12 D	3.39 ± 0.08\u00B0C	2.99 ± 0.08 B

Effect of bacterial inoculation on soil available P collected at 35 DAS and 65 DAS and during harvesting stage, plant P at 35 DAS and 65 DAS in a wheat pot experiment at Faisalabad. Values represented are an average of three replicates. ± indicates standard deviation (SD). Means with significant differences (*p* < 0.05) among treatments are represented by different letters. Control 100%: Recommended dose of DAP without inoculation, Control 80%: 20% less DAP than the recommended dose without inoculation, Con-1: Consortium-1+ 80% DAP. Con-2: Consortium-2 + 80% DAP. Con-3: Consortium-3 + 80% DAP.

### Microcosm studies for solubilization of RP in BOP formulations

**Figure 3:**
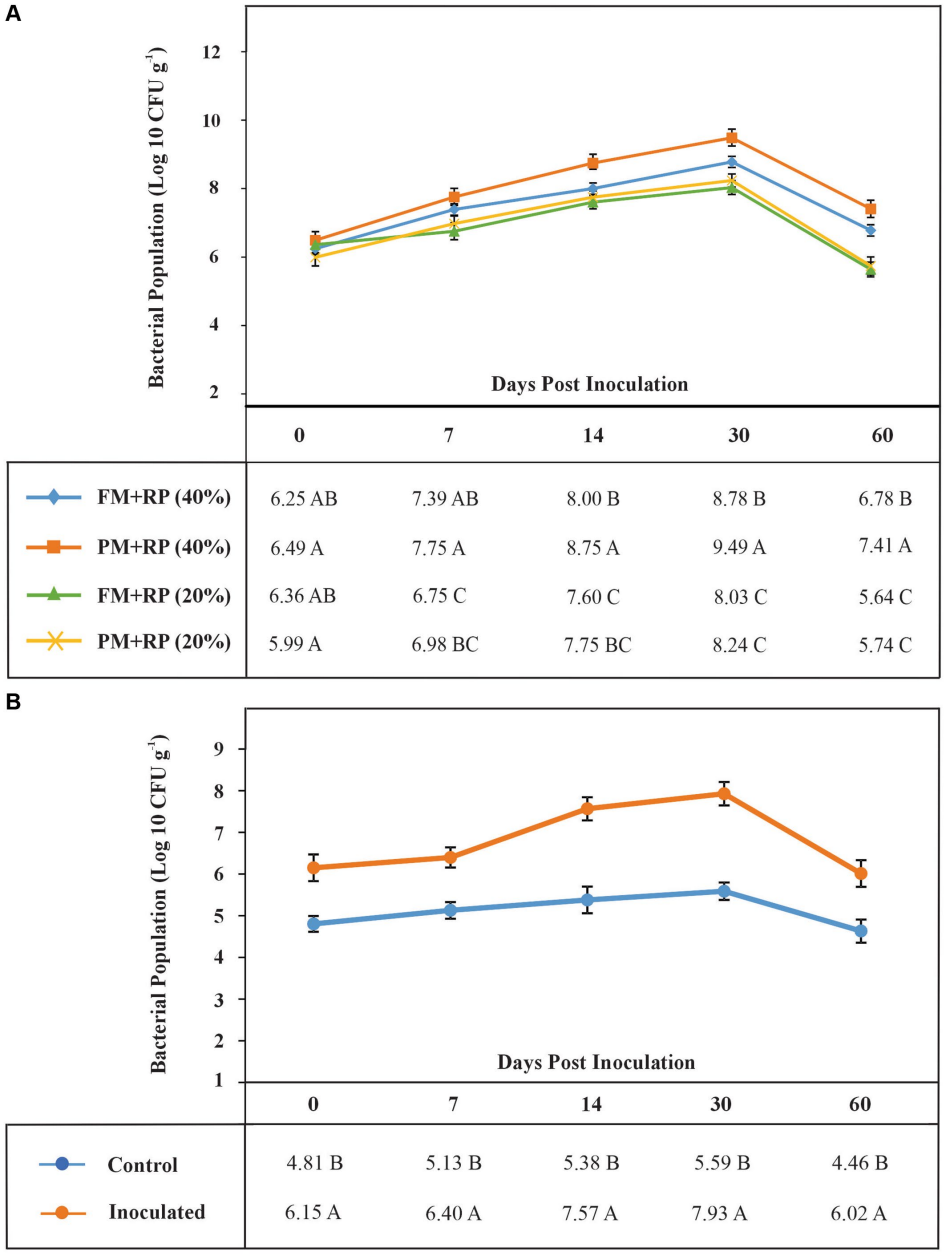
The shelf life of bioformulations to assess the survival of inoculated PSB in different treatments **(A)**. Each point represents decimal logarithmic of viable cells g^−1^ carrier material and it is the mean value of three replicas. The ANOVA has been performed within each sampling date, within each column in data table, and the values followed by the same letter, within each column, are not significantly different at *p*-value of <0.05 in the LSD test. The shelf life of bioformulations to assess the survival of inoculated PSB in BOP **(B)**. Each point represents decimal logarithmic of viable cells g^−1^ BOP, and it is the mean value of three replicas. The ANOVA has been performed within each sampling date, within each column in data table.

Plant material-based BOP formulation increased P (14%) content at 40% moisture level as compared with the uninoculated control (Table 4). Plant material-based BOP also had more viable count (3.5 × 10^9^ CFU) as compared with filter mud-based BOP (Figure 3A).

**Table 4:** Total P content in BOP at different time intervals.

Total P (%)			
Sr.no	Treatments	30-DPI	60-DPI
1	Filter Mud + Rock Phosphate (40%)	8.01 ± 0.02 C	8.65 ± 0.03 C
2	Plant Material + Rock Phosphate (40%)	10.8 ± 0.06 A	14.21 ± 0.05 A
3	Filter Mud + Rock Phosphate (20%)	7.65 ± 0.01 D	8.03 ± 0.01 D
4	Plant Material + Rock Phosphate (20%)	8.9 ± 0.03 B	13.29 ± 0.07 B

Detection of total Phosphorous in microcosmic study of BOP experiment at 30- and 60-days post inoculation. Values represented are an average of three replicates. ± indicates standard deviation (SD). Means with significant differences (*p* < 0.05) among treatments are represented by different letters. FM; Filter mud; PM: Plant Material; RP: Rock Phosphate; 40%: 40 percent moisture content and 20%: 20 percent moisture content.

### Development of BOP under natural environmental conditions

Based on microcosm results, plant-based BOP was formulated under natural environmental conditions. The survival of heat-tolerant PSB inoculated in BOP was evaluated at 0 h on 7 DPI, 14 DPI, and 30 DPI by the viable count method (Figure 3B). All inoculated strains were observed during these intervals. The single isolated colonies of ZR2 and CH4 were observed on 14 DPI, while ZR1, ZR3, and ZR4 were observed on 30 DPI.

Nitrogen (N), potassium (K), electrical conductivity, moisture content, and pH of both the inoculated and uninoculated treatments of BOP were analyzed using a nutrient analyzer (Ramanaiah et al., 2021; Table 5). The nutrient content of both the inoculated and uninoculated treatments of BOP were analyzed using a nutrient analyzer, where the results showed that nitrogen (39%) and potassium (45%) contents were significantly high in inoculated BOP sample as compared with the uninoculated control. Meanwhile, the P content was significantly higher (8%) in inoculated BOP as compared with the control (Table 5).

**Table 5:** Physicochemical and nutritional parameters of BOP.

Treatments	N (mg kg^−1^)	K (mg kg^−1^)	pH	Moisture content %	EC (μS/cm)	P (ppm)	Total P (%)
Control	22.33 ± 1.15 B	70.00 ± 3.46 B	7.00 ± 0.04 B	43.73 ± 2.12 A	448.33 ± 12.42 B	112.52 B	6.21 B
BOP	31 ± 1.00 A	101.67 ± 0.58 A	7.04 ± 0.08 A	44.20 ± 2.35 A	614.33 ± 3.79 A	158.54 A	8.01 A

Detection of nitrogen, phosphorous, potassium, pH, moisture content, and EC at 60 DPI of BOP Experiment. Values represented are an average of three replicates. ± indicates standard deviation (SD). Means with significant differences (*p* < 0.05) among treatments are represented by different letters. Control: Plant Material+ Rock Phosphate +Molasses, BOP: Plant Material+ Rock Phosphate + PSB.

### Evaluation of BOP for wheat yield parameters

*In planta* evaluation of BOP on wheat growth parameters was performed in a pot experiment under net house conditions at NIBGE, Faisalabad. Plants were uprooted at different growth stages, and different plant growth characteristics such as root length, shoot length, and plant biomass (fresh weight and dry weight) were assessed (Table 6). The results revealed that the BOP had a promising influence on many plant growth parameters. Physiochemical properties of the soil collected from pots at 35 days after sowing (DAS) were also evaluated using a nutrient analyzer (Table 7). Chlorophyll content was also evaluated at 35 DAS using a SPAD meter (Table 6).

**Table 6:** Effect of BOP on plant growth parameters, yield, and chlorophyll content at different growth stages in the pot experiment under net house conditions.

At 35 DAS						At 65 DAS				At harvest stage					
Treatments	Root length (cm)	Shoot length (cm)	Fresh weight (g)	Dry weight (g)	Chlorophyll	Root length (cm)	Shoot length (cm)	Fresh weight (g)	Dry weight (g)	Plant height (cm)	Tillers	100 Grain weight (g)	Plant biomass (g)	Grain yield (g / plant)	Viable count
Control 100%	6.87 ± 1.72 A	35.87 ± 1.99 A	3.00 ± 0.17 AB	0.43 ± 0.06 B	83.09 ± 1.40 AB	14.00 ± 2.65 AB	61.67 ± 2.52 B	19.83 ± 2.80 B	5.67 ± 0.40 B	70.33 ± 3.43 B	5.00 ± 0.71 B	4.44 ± 0.13 B	14.58 ± 0.66 B	8.42 ± 0.40 B	3.5 × 10^7^
Control 80%	5.97 ± 1.42 A	39.77 ± 1.50 B	2.53 ± 0.32 B	0.38 ± 0.04 B	82.43 ± 0.86 B	12.33 ± 2.08 B	56.67 ± 2.52 C	15.00 ± 2.00 C	3.87 ± 0.57 C	65.22 ± 4.60 C	3.89 ± 0.60\ C	3.46 ± 0.15 C	12.41 ± 0.47 C	5.02 ± 0.47 C	3.07 × 10^7^
BOP	7.93 ± 1.48 A	36.07 ± 1.12 A	3.30 ± 0.30 A	0.57 ± 0.06 A	84.18 ± 1.05 A	19.67 ± 4.51 A	69.33 ± 1.53 A	26.00 ± 2.00 A	6.97 ± 0.55 A	79.67 ± 3.97 A	5.78 ± 0.44 A	4.92 ± 0.43 A	17.84 ± 0.48 A	9.76 ± 0.39 A	3.95 × 10^7^

Effect of bacterial inoculation on various wheat growth parameters at 35 DAS (1) and 65 DAS (2) and during harvesting stage (3) in a pot experiment in Faisalabad. Values represented are an average of three replicates. ± indicates standard deviation (SD). Means with significant differences (*p* < 0.05) among treatments are represented by different letters. Control 100%: Recommended dose of DAP without inoculation, Control 80%: 20% less DAP than the recommended dose without inoculation, BOP: Plant material +Rock Phosphate+ Consortium-2 + 80% DAP.

**Table 7:** Effect of BOP on physiochemical parameters of soil in the pot experiment under net house conditions.

Treatments	N (mg kg^−1^)	K (mg kg^−1^)	pH	Moisture content %	EC μS/cm
Control 100%	13.22 ± 1.9 B	44.67 ± 7.9 B	7.42 ± 0.54 B	11.71 ± 2.76 A	270.11 ± 20.52 B
Control 80%	11 ± 1.42 C	36.22 ± 5.9 C	7.22 ± 0.36 B	10 ± 1.90 A	239.56 ± 19.45 C
BOP	16.33 ± 1.7 A	60 ± 5.05 A	8 ± 0.19 A	12.27 ± 2.65 A	295 ± 16.85 A

Effect of bacterial inoculation on various physiochemical parameters of soil collected at 35 DAS in a pot experiment in Faisalabad. Values represented are an average of three replicates. ± indicates standard deviation (SD). Means with significant differences (*p* < 0.05) among treatments are represented by different letters. Control 100%: Recommended dose of DAP without inoculation, Control 80%: 20% less DAP than the recommended dose without inoculation, BOP: Plant material +Rock Phosphate+ Consortium-2 + 80% DAP.

**Figure 4:**
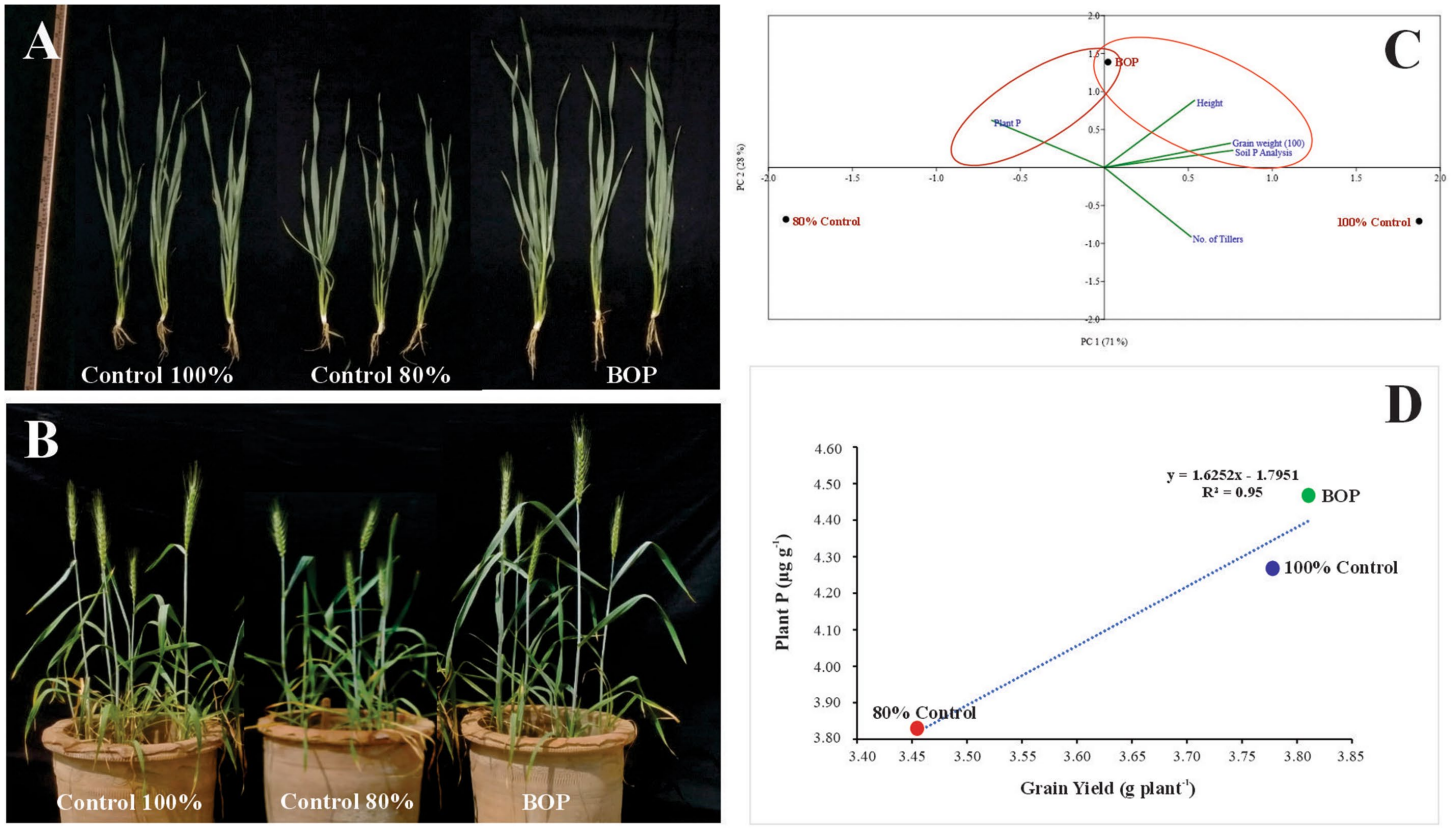
Effect of BOP on vegetative growth of wheat **(A)**, Effect of BOP on wheat plant growth and yield **(B)**, Principal component analysis **(C)**, Regression analysis **(D)**. Control 100%: Recommended dose of DAP without inoculation; Control 80%: 20% less DAP than the recommended dose without inoculation; BOP: Plant material +Rock phosphate+ Consortium-2 + 80% DAP.

At 35 DAS, 65 DAS, and harvesting stage, soil available P was evaluated for inoculated pots and control. At 35 DAS and harvesting stage, plant P was also evaluated (Table 8). The results revealed that soil amended with plant material-based BOP (comprised of plant material, RP, and consortium-2) had a promising influence on many plant growth parameters. Soil physicochemical parameters were determined, such as available P content at 10.72 ppm at 65 DAS of the BOP-amended soil. In the BOP-inoculated treatment, maximum yield was observed in pots increased grain yield (9.7 g plant^−1^) in comparison to both uninoculated 80% (5 g plant^−1^) and 100% control (8.4 g plant^−1^). Principle component analysis (PCA) indicates a positive correlation between BOP and grain weight, height, and soil available P as compared with both 100 and 80% controls. The two principal components contributed up to 99% toward variance on the x-axis (PC 1 = 71%) and y-axis (PC 2 = 25%). No parameter was found to have a negative effect on the application of BOP. Regression analysis further indicated a positive correlation between yield and plant P in pots with BOP (Figure 4).

**Table 8:** Effect of BOP on soil, plant, and grain P in the pot experiment under net house conditions.

	Soil available P (ppm)			Plant P (%)		Grain P (%)
Treatments	At 35 DAS	At 65 DAS	At harvest stage	At 35 DAS	At 65 DAS	At harvest stage
Control 100%	11.52 ± 0.17 A	9.51 ± 0.14 B	9.14 ± 0.10 B	3.80 ± 0.06 B	3.31 ± 0.06 B	4.268 ± 0.04 B
Control 80%	9.67 ± 0.14 C	7.01 ± 0.08 C	6.95 ± 0.14 C	3.39 ± 0.07 C	2.88 ± 0.08 C	3.8 ± 0.04 **C**
BOP	10.50 ± 0.29 B	10.72 ± 0.16 A	10.00 ± 0.10 A	4.09 ± 0.06 A	3.85 ± 0.08 A	4.468 ± 0.04 A

Effect of different BOP by soil amendment on available P of soil collected at 35 DAS and 65 DAS and during harvesting stage, plant P at 65 DAS, and grain P at harvesting stage in a wheat pot experiment in Faisalabad. Values represented are an average of three replicates. ± indicates standard deviation (SD). Means with significant differences (*p* < 0.05) among treatments are represented by different letters. Control 100%: Recommended dose of DAP without inoculation, Control 80%: 20% less DAP than the recommended dose without inoculation, BOP: Plant material +Rock Phosphate+ Consortium-2 + 80% DAP.

**Figure 5:**
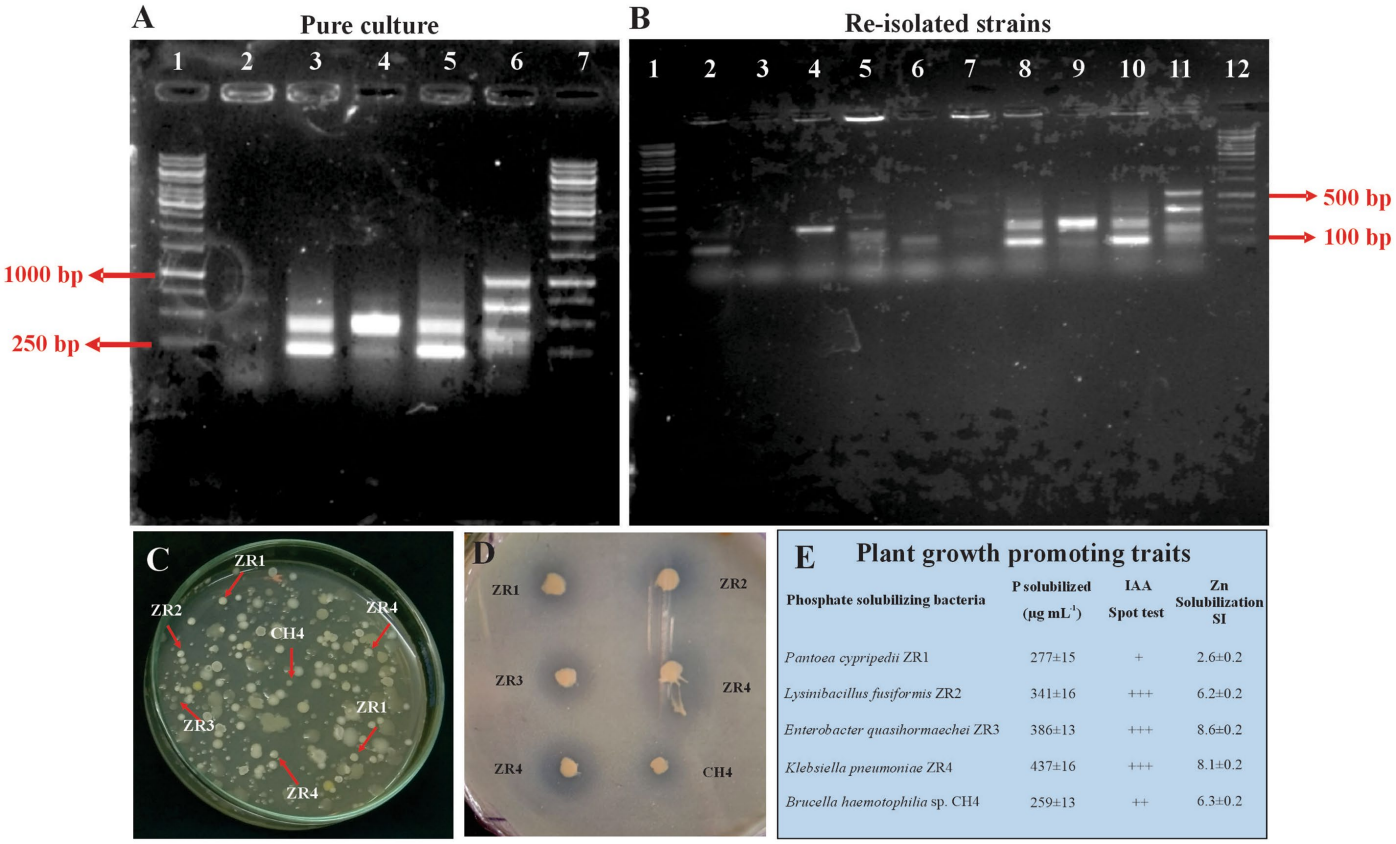
Re-isolation of inoculated PSB colonies. Photograph of gel indicates BOX-PCR patterns of pure culture of PSB from wheat rhizosphere, 1: 1Kb DNA ladder; 2: ZR1, 3: ZR2, 4: ZR3, 5: ZR4, 6: CH4 **(A)**. Photograph of gel indicates re-isolated colonies of PSB that are morphologically similar to the inoculated consortium-2 PSB, 1: 1Kb DNA ladder, 2–5: Non-specific colony, 6: Re-isolated ZR1 colony, 7: Re-isolated ZR2 colony, 8: Re-isolated ZR3 colony, 9: Re-isolated ZR4 colony, 10: Re-isolated CH4 colony, 11: Non-specific colony **(B)**. Plate showing re-isolated colonies of ZR1, ZR2, ZR3, ZR4, and CH4 **(C)**. NBRIP Plate showing solubilization zones formed by re-isolated colonies of ZR1, ZR2, ZR3, ZR4, and CH4 **(D)**. Plant growth-promoting attributes of re-isolated PSB strains for the confirmation of inoculated PSB: P solubilization was detected quantitatively by the molybdenum blue method, IAA production indicated by pink coloration upon reaction with Salkowski’s reagent, and Zinc solubilization was confirmed by the formation of halo zone on an agar plate added with ZnO. Values are an average of three biological replicates **(E)**.

The viable count method ensured the survival of inoculated PSB. Morphological characteristics of the inoculated PSB facilitated in the detection of inoculated rhizospheric bacteria, demonstrating their rhizosphere competence. BOX-PCR confirmed the inoculated phosphate-solubilizing bacteria. BOX-PCR of re-isolated colonies was found identical to that of pure cultures (Figure 5).

## Discussion

Phosphorous is the second most essential and limiting macronutrient for plant growth, which is naturally found in the soil in insoluble forms (Bamagoos et al., 2021). Plant roots absorb P in the forms of H_2_PO_4_^−^ and HPO_4_^2−^ depending on soil pH. As a result, a small proportion of soil P (0.1%) is readily accessible to plants. Phosphorus deficiency prevents normal plant growth if it is not supplied in enough quantities by the soil or fertilizers. Thus, P deficiency can cause up to 15% reduction in crop yield (Shenoy and Kalagudi, 2005). Phosphatic fertilizers are commonly used to address P deficiency in the soil. Modern agricultural practices have emphasized the massive use of chemicals, resulting in enhanced grain yield in various countries over the last decades. However, continual and excessive use of chemical fertilizers led to a decline in soil fertility and environmental pollution. Consequently, the discovery of an efficient method for the formulation of an eco-friendly, low cost, and competent organic fertilizer is an essential task for the world’s future agricultural development (Elhaissoufi et al., 2022). Therefore, keeping in view the importance of an integrated nutrient management approach, in the present study, heat-tolerant P-solubilizing bacteria were integrated with RP along with organic amendments, to form BOP for sustainable production of wheat.

Phosphate-solubilizing bacteria were isolated from rhizospheric soils of wheat collected from several regions of South Punjab, Pakistan. For the isolation of heat-tolerant PSB, soil samples were enriched in NBRIP broth medium at different temperatures (45° C to 60° C). Out of 79 isolates, 21 colonies showed phosphate-solubilizing activity. A higher number of P solubilizers were obtained from Mankera soils at 45° C in second NBRIP enrichment, while no PSB was found at 60° C.

These PSB can solubilize TCP in the range of 291 μg ml − ^1^ to 454 μg ml − ^1^ with a decrease in pH from 7 to 4.5 at 45° C and 278 μg ml − ^1^ to 421 μg ml − ^1^ at 50° C with a decrease in pH from 7 to 4.6. The PSB strain ZR4 showed maximum phosphate solubilization activity (454 μg ml − ^1^) with the highest solubilizing index (SI: 3.75), followed by ZR3 (SI: 3.2) and ZR2 (SI: 3.00), whereas the least amount of P was solubilized by ZR1 (291 μg ml − ^1^). A decrease in pH of the medium was observed with the inoculation of different PSB at 45° C and 50° C (Figure 2). The production of organic acid is the principal mechanism used by PSB for inorganic phosphate solubilization. The production of these organic acids by the phosphate-solubilization process result in a reduction in pH of the medium (Gyaneshwar et al., 1999; Puente et al., 2004; Khan et al., 2014).

Light microscopy and gram staining showed that all PSB (ZR1, ZR2, ZR3, ZR4, and ZR5) were gram-negative. All the studied PSB were rod shaped. Only ZR1 and ZR3 were motile. The 16S rRNA gene sequencing of PSB identified some bacteria, such as *P. cypripedii*, *L. fusiformis*, *E. quasihormaechei, K. pneumoniae,* and *K. quasi*var*iicola.* The identified bacterial genera have been reported as plant growth-promoting bacteria (Kumar et al., 2014; Chauhan et al., 2015; Lamizadeh et al., 2016; Gontia-Mishra et al., 2017a; Kusale et al., 2021; Passera et al., 2021; Belaouni et al., 2022).

The selected PSB were further evaluated for different plant growth-promoting attributes. PSB (ZR2, ZR3, and ZR4) were involved in the production of indole acetic acid. The production of IAA enhances root growth parameters such as root length surface area and, consequently, improves the nutrient concentration in soil and its efficacy (Etesami et al., 2015). Some of the bacteria showed nitrogen-fixing ability (PSB: ZR1, ZR2, ZR4, and ZR5) as detected by bacterial growth and change in color of Nitrogen Free Malate (NFM) medium from green to blue in glass vials. Bacterial nitrogen fixation may help to enhance wheat N content. Previous studies also reported the application of N fixers in the wheat, enhancing plant growth and crop yield (Majeed et al., 2015). Zinc-solubilizing activity was shown by ZR1, ZR2, ZR3, ZR4, and ZR5. The solubilization index ranged from 2.8 to 8.75 in a plate assay. ZR3 showed the highest solubilizing index (8.75). Zinc is another important nutrient which is required in small amount for proper plant growth and regulation of essential metabolic reactions. It also forms insoluble complexes that are inaccessible to plants. This fixed zinc can be solubilized with the help of phosphate-solubilizing microorganisms by the production of protons and chelated compounds. The most abundant zinc solubilizers reported are *Bacillus* sp. (Gandhi and Muralidharan, 2016; Sunithakumari et al., 2016; Gontia-Mishra et al., 2017b). PSB, i.e., *Enterobacter* sp. ZR3 and *Klebsiella* sp. ZR5 with zinc-solubilizing activity may be able to solubilize many nutrients in stressed or nutrient-deficient soils. PSB (*Lysinibacillus* sp. ZR2 and *Klebsiella* sp. ZR4) showed the production of ACC deaminase that helps to mitigate harmful effects on plant growth and reduce various stresses. Studies have reported that using ACC deaminase-producing bacteria may help lower the severity of stress (Glick, 2014).

In addition to having multiple plant growth-promoting traits, these bacteria were capable to produce EPS that has a high potential to ameliorate stresses in plants. Thin layer chromatographic analysis showed that the isolate *P. cypripedii* ZR1 produced EPS using sucrose as a substrate. *P. cypripedii* 4A has also been reported to be a potential producer of extracellular polysaccharides (Sazonova et al., 2021). Microbial EPS has numerous significant physiological functions and commercial uses derived from their roles in nature. These high-molecular weight polymers play a key role in various cellular functions such as cell protection from antimicrobial agents, freezing and dehydration, adherence to surfaces, other organisms and biofilm production, biofilm inhibition, and storage of reserve carbon sources (Silvi et al., 2013).

For the evaluation of thermo-tolerant PSB on wheat crop, three potential consortia were developed. In the pot experiment, these three potential consortia were evaluated on wheat variety AKBAR-2019 under net house conditions. The results showed that the consortium-2 had a favorable impact on every growth parameter. Maximum yield was observed in pots inoculated with the consortium-2 including *Pantoea* sp. ZR1, *L. fusiformis* ZR2, *Enterobacter* sp. ZR3, *K. pneumoniae* sp. ZR4, and *B. haematophilia* sp. CH4. These PSB enhanced grain yield (9.63 g plant^−1^) as compared with 80% uninoculated control. Soil available P of the consortium-2 treatment was significantly higher (10 μg g^−1^) as compared with uninoculated 80% control during harvesting. The inoculation of PSB in wheat-grown soil has been reported to increase the amount of available P as compared with the uninoculated control (Yahya et al., 2021). Previous studies indicated an increased amount of available P and enhanced wheat plant development in soils inoculated with phosphate-solubilizing consortia (Boubekri et al., 2021).

Furthermore, a microcosm study was conducted to evaluate the effect of consortium-2 on the formulation of BOP. For this purpose, consortium-2 was inoculated in reagent bottles containing RP and different sources of organic matter, i.e., filter mud and plant material as a substrate. Plant material-based BOP formulation increased P (14%) at 40% moisture level as compared with the uninoculated control. Plant material-based BOP had more viable count (3.5 × 10^9^ CFU) as compared with filter-mud based BOP. BOP is reported to enhance nutrient availability by providing an appropriate habitat for beneficial rhizospheric bacteria, and it also enriches soil organic matter. The bacteria are directly involved in making nutrients available in soil for plant uptake (Singh and Reddy, 2011; Anand et al., 2016).

In the present study, BOP was further developed and evaluated under natural environmental conditions in microplots. The survival of heat-tolerant PSB inoculated in BOP sample was evaluated up to 60 DPI by the viable count method. The nutrient contents of both inoculated and uninoculated treatments of BOP were analyzed by a nutrient analyzer, where the results showed significant increase in nitrogen (39%) and potassium (45%) contents of inoculated BOP sample as compared with the uninoculated control, whereas the P content was significantly higher (8%) in inoculated BOP as compared with the control. Studies showed the application of bio-organic fertilizer to improve soil N, P, K, and S concentrations compared with the non-treated control (Naher et al., 2021). These improved soil nutrient contents lead to improved soil organic matter and soil fertility, resulting in increased crop yield.

The effect of BOP on wheat growth parameters and phosphorous uptake was further evaluated on wheat variety AKBAR-2019 in a pot experiment under net house conditions. The results revealed that soil amended with plant-based BOP comprised of plant material, RP, and consortium-2 had a promising influence on many plant growth parameters; soil physicochemical parameters were enhanced, including available P content of 10.72 ppm at 65 DAS. Maximum yield was observed in pots with this treatment increased grain yield (9.7 g plant^−1^) as compared with both uninoculated 80% (5 g plant^−1^) and 100% control (8 g plant^−1^). The viable count method ensured the survival of inoculated PSB. Morphological characteristics of the inoculated PSB facilitated the detection of inoculated rhizospheric bacteria, demonstrating their rhizosphere competence. BOX-PCR revealed that these were rhizosphere-competent PSB. Moreover, plant growth-promoting traits of re-isolated bacteria in comparison with those of the pure culture of inoculated strain indicated the persistence of inoculated PSB (Figure 5).

Thus, the best remedy for soil nutrient management is a combination of both biofertilizers and organic fertilizers with the reduced application of chemical fertilizers, which ensures continuous supply of nutrients to plants, while organic amendments improve soil structure and soil buffering capacity. This has laid a solid foundation for further judicious combination of fertilizers to optimize nutrient availability, enhance crop yield, and maintain soil health in the long term. The study can further be evaluated for better cropping on a larger scale.

## Conclusion

Heat-tolerant multi-functional phosphate-solubilizing microbes positively improved wheat growth parameters. The inoculation of these microbes into plant material accelerates its decomposition during the preparation of biofertilizers with enhanced phosphorus content. The formulation and application of BOP comprised of plant material, RP, and heat-tolerant PSB can substantially improve wheat yield and P nutrition as compared with the control under net house conditions. Hence, the application of BOP with reduced application dose of DAP is recommended for obtaining optimum and quality production of wheat in calcareous soils. However, these findings need to be further verified under diverse agro-climatic conditions in long-term field trials before formulating countrywide recommendations.

## Data availability statement

The datasets presented in this study can be found in online repositories. The names of the repository/repositories and accession number(s) can be found in the article/Supplementary material.

## Author contributions

ZA: Conceptualization, Data curation, Formal analysis, Investigation, Methodology, Software, Validation, Visualization, Writing – original draft. MY: Conceptualization, Data curation, Formal analysis, Investigation, Methodology, Software, Supervision, Validation, Visualization, Writing – original draft, Writing – review & editing. HH: Formal analysis, Writing – review & editing. ST: Formal analysis, Methodology, Writing – review & editing. SJ: Formal analysis, Software, Writing – review & editing. SK: Conceptualization, Data curation, Methodology, Project administration, Supervision, Writing – review & editing. SY: Conceptualization, Data curation, Formal analysis, Funding acquisition, Investigation, Methodology, Project administration, Resources, Software, Supervision, Validation, Visualization, Writing – original draft, Writing – review & editing.

## Footnotes

^1^ http://www.ncbi.nlm.nih.gov/

## References

[ref1] Aimen A. Basit A. Bashir S. Aslam Z. Shahid M. F. Amjad S.. (2022). Sustainable phosphorous management in two different soil series of Pakistan by evaluating dynamics of phosphatic fertilizer source. Saudi J. Biolog. Sci. 29, 255–260. doi: 10.1016/j.sjbs.2021.08.086, PMID: 35002416 PMC8717160

[ref2] Anand K. Kumari B. Mallick M. (2016). Phosphate solubilizing microbes: an effective and alternative approach as biofertilizers. Int J Pharm Pharm Sci 8, 37–40.

[ref3] Andersen J. M. (1976). An ignition method for determination of total phosphorus in lake sediments. Water Res. 10, 329–331. doi: 10.1016/0043-1354(76)90175-5

[ref4] Ashraf N. Anwar M. A. Akhtar K. Yasmin S. Khaliq S. (2022). Draft genome sequence of streptomyces sp. strain r1, isolated from water canal sediments, possessing antimicrobial and plant growth promoting capabilities. Microbiol. Resource Announcements 11, e00725–e00722. doi: 10.1128/mra.00725-22, PMID: 35972253 PMC9476928

[ref5] Ashraf N. Bechthold A. Anwar M. A. Khaliq S. (2020). Draft genome sequence of *Streptomyces* sp. strain br123, endowed with broad-spectrum antimicrobial potential. Microbiol. Resource Announcements 9:10.1128/mra.00972-20. doi: 10.1128/MRA.00972-20, PMID: 33033135 PMC7545289

[ref6] Azaroual S. E. Hazzoumi Z. Mernissi N. E. Aasfar A. Meftah Kadmiri I. Bouizgarne B. (2020). Role of inorganic phosphate solubilizing bacilli isolated from moroccan phosphate rock mine and rhizosphere soils in wheat (*Triticum aestivum L*) phosphorus uptake. Curr. Microbiol. 77, 2391–2404. doi: 10.1007/s00284-020-02046-8 32468184

[ref7] Bamagoos A. Alharby H. Fahad S. (2021). Biochar coupling with phosphorus fertilization modifies antioxidant activity, osmolyte accumulation and reactive oxygen species synthesis in the leaves and xylem sap of rice cultivars under high-temperature stress. Physiol. Mol. Biol. Plants 27, 2083–2100. doi: 10.1007/s12298-021-01062-7, PMID: 34629780 PMC8484400

[ref9003] Basheer A. Zaheer A. Qaisrani M. M. Rasul G. Yasmin S. Mirza M. S. (2016). Development of DNA markers for detection of inoculated bacteria in the rhizosphere of wheat (Triticum aestivum L.). Pak. J. Agric. Sci. 53. doi: 10.21162/PAKJAS/16.2324

[ref8] Belaouni H. A. Bendaha M. E. A. Benattia H. Medouh M. Berini K. I. Ben Ahmed S.. (2022). Alleviation of salt stress in winter wheat by *Pantoea* spp. endophytes isolated from spontaneous desert plants of the Sahara. Arch. Phytopathol. Plant Protect. 55, 2334–2355. doi: 10.1080/03235408.2023.2165431

[ref9] Boubekri K. Soumare A. Mardad I. Lyamlouli K. Hafidi M. Ouhdouch Y.. (2021). The screening of potassium- and phosphate-solubilizing Actinobacteria and the assessment of their ability to promote wheat growth parameters. Microorganisms 9:470. doi: 10.3390/microorganisms9030470, PMID: 33668691 PMC7996285

[ref10] Bouizgarne B. (2022). Phosphate-solubilizing actinomycetes as biofertilizers and biopesticides: bioformulations for sustainable agriculture. Microbial BioTechnol. Sustain. Agricul. 1:13. doi: 10.1007/978-981-16-4843-4_13

[ref11] Bouizgarne B. Bakki M. Boutasknit A. Banane B. El Ouarrat H. Ait El Maalem S.. (2023). Phosphate and potash solubilizing bacteria from Moroccan phosphate mine showing antagonism to bacterial canker agent and inducing effective tomato growth promotion. Front. Plant Sci. 14:970382. doi: 10.3389/fpls.2023.970382, PMID: 36968412 PMC10030999

[ref12] Chauhan H. Bagyaraj D. J. Selvakumar G. Sundaram S. P. (2015). Novel plant growth promoting rhizobacteria—prospects and potential. Appl. Soil Ecol. 95, 38–53. doi: 10.1016/j.apsoil.2015.05.011

[ref13] Chung H. Park M. Madhaiyan M. Seshadri S. Song J. Cho H.. (2005). Isolation and characterization of phosphate solubilizing bacteria from the rhizosphere of crop plants of Korea. Soil Biol. Biochem. 37, 1970–1974. doi: 10.1016/j.soilbio.2005.02.025

[ref14] Cicek H. Bhullar G. S. Mandloi L. S. Andres C. Riar A. S. (2020). Partial acidulation of rock phosphate for increased productivity in organic and smallholder farming. Sustain. For. 12:607. doi: 10.3390/su12020607

[ref15] De Oliveira Mendes G. Murta H. M. Valadares R. V. Da Silveira W. B. Da Silva I. R. Costa M. D. (2020). Oxalic acid is more efficient than sulfuric acid for rock phosphate solubilization. Miner. Eng. 155:106458. doi: 10.1016/j.mineng.2020.106458

[ref16] Ditta A. Imtiaz M. Mehmood S. Rizwan M. S. Mubeen F. Aziz O.. (2018). Rock phosphate-enriched organic fertilizer with phosphate-solubilizing microorganisms improves nodulation, growth, and yield of legumes. Commun. Soil Sci. Plant Anal. 49, 2715–2725. doi: 10.1080/00103624.2018.1538374

[ref17] Elhaissoufi W. Ghoulam C. Barakat A. Zeroual Y. Bargaz A. (2022). Phosphate bacterial solubilization: a key rhizosphere driving force enabling higher P use efficiency and crop productivity. J. Adv. Res. 38, 13–28. doi: 10.1016/j.jare.2021.08.014, PMID: 35572398 PMC9091742

[ref18] Etesami H. Alikhani H. A. Hosseini H. M. (2015). Indole-3-acetic acid (IAA) production trait, a useful screening to select endophytic and rhizosphere competent bacteria for rice growth promoting agents. MethodsX 2, 72–78. doi: 10.1016/j.mex.2015.02.008, PMID: 26150974 PMC4487705

[ref19] Gandhi A. Muralidharan G. (2016). Assessment of zinc solubilizing potentiality of Acinetobacter sp. isolated from rice rhizosphere. Eur. J. Soil Biol. 76, 1–8. doi: 10.1016/j.ejsobi.2016.06.006

[ref20] Gao C. El-Sawah A. M. Ali D. F. I. Alhaj Hamoud Y. Shaghaleh H. Sheteiwy M. S. (2020). The integration of bio and organic fertilizers improve plant growth, grain yield, quality and metabolism of hybrid maize (*Zea mays L*.). Agronomy 10:319. doi: 10.3390/agronomy10030319

[ref21] Glick B. R. (2014). Bacteria with ACC deaminase can promote plant growth and help to feed the world. Microbiol. Res. 169, 30–39. doi: 10.1016/j.micres.2013.09.009, PMID: 24095256

[ref22] Goldstein A. H. Rogers R. D. Mead G. (1993). Mining by microbe. Bio/Technology 11, 1250–1254. doi: 10.1038/nbt1193-1250

[ref23] Gontia-Mishra I. Sapre S. Kachare S. Tiwari S. (2017a). Molecular diversity of 1-aminocyclopropane-1-carboxylate (ACC) deaminase producing PGPR from wheat (*Triticum aestivum L*.) rhizosphere. Plant Soil 414, 213–227. doi: 10.1007/s11104-016-3119-3

[ref24] Gontia-Mishra I. Sapre S. Tiwari S. (2017b). Zinc solubilizing bacteria from the rhizosphere of rice as prospective modulator of zinc biofortification in rice. Rhizosphere 3, 185–190. doi: 10.1016/j.rhisph.2017.04.013

[ref25] Gordon S. A. Weber R. P. (1951). Colorimetric estimation of indoleacetic acid. Plant Physiol. 26, 192–195. doi: 10.1104/pp.26.1.192, PMID: 16654351 PMC437633

[ref26] Gulati A. Sharma N. Vyas P. Sood S. Rahi P. Pathania V.. (2010). Organic acid production and plant growth promotion as a function of phosphate solubilization by Acinetobacter rhizosphaerae strain BIHB 723 isolated from the cold deserts of the trans-Himalayas. Arch. Microbiol. 192, 975–983. doi: 10.1007/s00203-010-0615-3, PMID: 20821196

[ref27] Gyaneshwar P. Parekh L. J. Archana G. Poole P. S. Collins M. D. Hutson R. A.. (1999). Involvement of a phosphate starvation inducible glucose dehydrogenase in soil phosphate solubilization by *Enterobacter asburiae*. FEMS Microbiol. Lett. 171, 223–229. doi: 10.1016/S0378-1097(99)00003-8

[ref28] Hardy R. F. Holsten R. D. Jackson E. K. Burns R. C. (1968). The acetylene-ethylene assay for N2 fixation: laboratory and field evaluation. Plant Physiol. 43, 1185–1207. doi: 10.1104/pp.43.8.1185, PMID: 16656902 PMC1086994

[ref29] Ibrahim M. Iqbal M. Tang Y.-T. Khan S. Guan D.-X. Li G. (2022). Phosphorus mobilization in plant-soil environments and inspired strategies for managing phosphorus: a review. Agronomy 12:2539. doi: 10.3390/agronomy12102539

[ref30] Irabor A. Mmbaga M. (2017). Evaluation of selected bacterial endophytes for biocontrol potential against phytophthora blight of bell pepper (*Capsicum annuum L*.). J Plant Pathol Microbiol 8:424. doi: 10.4172/2157-7471.1000424

[ref31] Kari A. Nagymáté Z. Romsics C. Vajna B. Kutasi J. Puspán I.. (2019). Monitoring of soil microbial inoculants and their impact on maize (*Zea mays* L.) rhizosphere using T-RFLP molecular fingerprint method. Appl. Soil Ecol. 138, 233–244. doi: 10.1016/j.apsoil.2019.03.010

[ref32] Khakwani A. Imran M. Ahmad I. Waqas R. Hussain S. Nadeem S. M.. (2017). Comparative efficacy of bio-organic and mineral phosphate on the growth, yield and economics of wheat (Triticum aestivum L.) grown by different methods. Commun. Soil Sci. Plant Anal. 48, 73–82. doi: 10.1080/00103624.2016.1253722

[ref33] Khan M. S. Zaidi A. Ahmad E. (2014). “Mechanism of phosphate solubilization and physiological functions of phosphate-solubilizing microorganisms” in Phosphate solubilizing microorganisms: Principles and application of Microphos technology. eds. Khan M. S. Zaidi A. Musarrat J. (Cham: Springer International Publishing).

[ref34] Kumar A. Maurya B. R. Raghuwanshi R. (2014). Isolation and characterization of PGPR and their effect on growth, yield and nutrient content in wheat (*Triticum aestivum L*.). Biocatal. Agric. Biotechnol. 3, 121–128. doi: 10.1016/j.bcab.2014.08.003

[ref35] Kusale S. P. Attar Y. C. Sayyed R. Z. Malek R. A. Ilyas N. Suriani N. L.. (2021). Production of plant beneficial and antioxidants metabolites by klebsiellavariicola under salinity stress. Molecules 26:1894. doi: 10.3390/molecules26071894, PMID: 33810565 PMC8037558

[ref36] Lambers H. Finnegan P. M. Laliberté E. Pearse S. J. Ryan M. H. Shane M. W.. (2011). Phosphorus nutrition of Proteaceae in severely phosphorus-impoverished soils: are there lessons to be learned for future crops? Plant Physiol. 156, 1058–1066. doi: 10.1104/pp.111.174318, PMID: 21498583 PMC3135942

[ref37] Lamizadeh E. Enayatizamir N. Motamedi H. (2016). Isolation and identification of plant growth-promoting rhizobacteria (PGPR) from the rhizosphere of sugarcane in saline and non-saline soil. Int. J. Curr. Microbiol. App. Sci. 5, 1072–1083. doi: 10.20546/ijcmas.2016.510.113

[ref38] Lasudee K. Tokuyama S. Lumyong S. Pathom-Aree W. (2018). Actinobacteria associated with arbuscular mycorrhizal *Funneliformis mosseae* spores, taxonomic characterization and their beneficial traits to plants: evidence obtained from mung bean (*Vigna radiata*) and Thai jasmine rice (*Oryza sativa*). Front. Microbiol. 9:1247. doi: 10.3389/fmicb.2018.01247, PMID: 29942292 PMC6004784

[ref39] Lynch J. P. (2007). Roots of the second green revolution. Aust. J. Bot. 55, 493–512. doi: 10.1071/BT06118

[ref40] Majeed A. Abbasi M. K. Hameed S. Imran A. Rahim N. (2015). Isolation and characterization of plant growth-promoting rhizobacteria from wheat rhizosphere and their effect on plant growth promotion. Front. Microbiol. 6:198. doi: 10.3389/fmicb.2015.00198, PMID: 25852661 PMC4362341

[ref42] Naher U. A. Biswas J. C. Maniruzzaman M. Khan F. H. Sarkar M. I. U. Jahan A.. (2021). Bio-organic fertilizer: a green technology to reduce synthetic N and P fertilizer for rice production. Front. Plant Sci. 12:602052. doi: 10.3389/fpls.2021.602052, PMID: 33833767 PMC8023392

[ref9001] Pakistan Standards and Quality Control Authority. (2021). Retrieved from https://www.psqca.com.pk/

[ref43] Passera A. Rossato M. Oliver J. S. Battelli G. Shahzad G.-I. R. Cosentino E.. (2021). Characterization of *Lysinibacillus fusiformis* strain S4C11: *in vitro, in planta*, and *in silico* analyses reveal a plant-beneficial microbe. Microbiol. Res. 244:126665. doi: 10.1016/j.micres.2020.126665, PMID: 33340794

[ref44] Paul D. Sinha S. N. (2017). Isolation and characterization of phosphate solubilizing bacterium *Pseudomonas aeruginosa* KUPSB12 with antibacterial potential from river ganga, India. Annals of Agrarian Sci. 15, 130–136. doi: 10.1016/j.aasci.2016.10.001

[ref45] Penrose D. M. Glick B. R. (2003). Methods for isolating and characterizing ACC deaminase-containing plant growth-promoting rhizobacteria. Physiol. Plant. 118, 10–15. doi: 10.1034/j.1399-3054.2003.00086.x, PMID: 12702008

[ref46] Plaxton W. C. Tran H. T. (2011). Metabolic adaptations of phosphate-starved plants. Plant Physiol. 156, 1006–1015. doi: 10.1104/pp.111.175281, PMID: 21562330 PMC3135920

[ref47] Puente M. E. Bashan Y. Li C. Y. Lebsky V. K. (2004). Microbial populations and activities in the rhizoplane of rock-weathering desert plants. I. Root colonization and weathering of igneous rocks. Plant Biol. (Stuttg.) 6, 629–642. doi: 10.1055/s-2004-821100, PMID: 15375735

[ref48] Ramanaiah S. Cordas C. M. Matias S. C. Reddy M. V. Leitão J. H. Fonseca L. P. (2021). Bioelectricity generation using long-term operated biocathode: RFLP based microbial diversity analysis. Biotechnol. Reports 32:e00693. doi: 10.1016/j.btre.2021.e00693, PMID: 34917493 PMC8666517

[ref49] Rasul M. Yasmin S. Yahya M. Breitkreuz C. Tarkka M. Reitz T. (2021). The wheat growth-promoting traits of *Ochrobactrum* and *Pantoea* species, responsible for solubilization of different P sources, are ensured by genes encoding enzymes of multiple P-releasing pathways. Microbiol. Res. 246:126703. doi: 10.1016/j.micres.2021.126703, PMID: 33482437

[ref50] Richardson A. E. Lynch J. P. Ryan P. R. Delhaize E. Smith F. A. Smith S. E.. (2011). Plant and microbial strategies to improve the phosphorus efficiency of agriculture. Plant Soil 349, 121–156. doi: 10.1007/s11104-011-0950-4

[ref51] Roset M. S. Ciocchini A. S. E. Ugalde R. A. Iñón De Iannino N. (2006). The *Brucella abortus* cyclic β-1, 2-glucan virulence factor is substituted with O-ester-linked succinyl residues. J. Bacteriol. 188, 5003–5013. doi: 10.1128/JB.00086-06, PMID: 16816173 PMC1539967

[ref52] Russell F. Biribo S. Selvaraj G. Oppedisano F. Warren S. Seduadua A.. (2006). As a bacterial culture medium, citrated sheep blood agar is a practical alternative to citrated human blood agar in laboratories of developing countries. J. Clin. Microbiol. 44, 3346–3351. doi: 10.1128/JCM.02631-05, PMID: 16954271 PMC1594681

[ref53] Sazonova O. I. Vetrova A. A. Gafarov A. B. Sharova M. V. Sokolov S. L. (2021). Identification and characteristics of *Pantoea cypripedii* 4A strain producing high molecular exopolysaccharide. Health, Food Biotechnol. 2, 70–80. doi: 10.36107/hfb.2020.i4.s90

[ref54] Senthilkumar N. A. M. Sankaranarayanan A. Senthilkumar (2021). Plant-microbe interactions. New York, United States: Springer.

[ref55] Sharma S. B. Sayyed R. Z. Trivedi M. H. Gobi T. A. (2013). Phosphate solubilizing microbes: sustainable approach for managing phosphorus deficiency in agricultural soils. Springerplus 2, 1–14. doi: 10.1186/2193-1801-2-587, PMID: 25674415 PMC4320215

[ref56] Shen J. Yuan L. Zhang J. Li H. Bai Z. Chen X.. (2011). Phosphorus dynamics: from soil to plant. Plant Physiol. 156, 997–1005. doi: 10.1104/pp.111.175232, PMID: 21571668 PMC3135930

[ref57] Shenoy V. V. Kalagudi G. M. (2005). Enhancing plant phosphorus use efficiency for sustainable cropping. Biotechnol. Adv. 23, 501–513. doi: 10.1016/j.biotechadv.2005.01.004 16140488

[ref58] Siedliska A. Baranowski P. Pastuszka-Woźniak J. Zubik M. Krzyszczak J. (2021). Identification of plant leaf phosphorus content at different growth stages based on hyperspectral reflectance. BMC Plant Biol. 21, 28–17. doi: 10.1186/s12870-020-02807-4, PMID: 33413120 PMC7792193

[ref59] Silvi S. Barghini P. Aquilanti A. Juarez-Jimenez B. Fenice M. (2013). Physiologic and metabolic characterization of a new marine isolate (BM39) of *Pantoea* sp. producing high levels of exopolysaccharide. Microb. Cell Factories 12:10. doi: 10.1186/1475-2859-12-10, PMID: 23360451 PMC3570286

[ref60] Singh H. Reddy M. S. (2011). Effect of inoculation with phosphate solubilizing fungus on growth and nutrient uptake of wheat and maize plants fertilized with rock phosphate in alkaline soils. Eur. J. Soil Biol. 47, 30–34. doi: 10.1016/j.ejsobi.2010.10.005

[ref61] Soltanpour P. Workman S. (1979). Modification of the NH_4_HCO_3_-DTPA soil test to omit carbon black. Commun. Soil Sci. Plant Anal. 10, 1411–1420. doi: 10.1080/00103627909366996

[ref62] Somasegaran P. Hoben H. J. (2012). Handbook for rhizobia: Methods in legume-Rhizobium technology. United States: Springer Science & Business Media.

[ref63] Suleman M. Yasmin S. Rasul M. Yahya M. Atta B. M. Mirza M. S. (2018). Phosphate solubilizing bacteria with glucose dehydrogenase gene for phosphorus uptake and beneficial effects on wheat. PLoS One 13:e0204408. doi: 10.1371/journal.pone.0204408, PMID: 30240432 PMC6150522

[ref64] Sunithakumari K. Devi S. N. P. Vasandha S. (2016). Zinc solubilizing bacterial isolates from the agricultural fields of Coimbatore, Tamil Nadu, India. Curr. Sci. 110, 196–205. doi: 10.18520/cs/v110/i2/196-205

[ref65] Tabbasum S. Akhtar M. Sarwar N. Tipu M. I. Ikram W. Ashraf A.. (2021). Relative effectiveness of phosphorus and potassium along with compost and organic acids on maize crop grown in calcareous soil: a multivariate analysis. J. Soil Sci. Plant Nutr. 21, 437–449. doi: 10.1007/s42729-020-00372-1

[ref66] Tahir M. Khalid U. Ijaz M. Shah G. M. Naeem M. A. Shahid M.. (2018). Combined application of bio-organic phosphate and phosphorus solubilizing bacteria (*Bacillus* strain MWT 14) improve the performance of bread wheat with low fertilizer input under an arid climate. Braz. J. Microbiol. 49, 15–24. doi: 10.1016/j.bjm.2017.11.005, PMID: 29728340 PMC6328723

[ref67] Tian J. Ge F. Zhang D. Deng S. Liu X. (2021). Roles of phosphate solubilizing microorganisms from managing soil phosphorus deficiency to mediating biogeochemical P cycle. Biology 10:158. doi: 10.3390/biology10020158, PMID: 33671192 PMC7922199

[ref68] Timofeeva A. Galyamova M. Sedykh S. (2022). Prospects for using phosphate-solubilizing microorganisms as natural fertilizers in agriculture. Plan. Theory 11:2119. doi: 10.3390/plants11162119, PMID: 36015422 PMC9414882

[ref69] United, S Tennessee Valley A. (1964). Superphosphate; its history, chemistry, and manufacture. Washington: Agricultural Research Service, U.S. Depart of Agriculture; for sale by the Supt. of Docs., U.S. Govt. Print. Off.

[ref70] Vance C. P. Uhde-Stone C. Allan D. L. (2003). Phosphorus acquisition and use: critical adaptations by plants for securing a nonrenewable resource. New Phytol. 157, 423–447. doi: 10.1046/j.1469-8137.2003.00695.x, PMID: 33873400

[ref71] Vassilev N. Mendes G. Costa M. Vassileva M. (2014). Biotechnological tools for enhancing microbial solubilization of insoluble inorganic phosphates. Geomicrobiol J. 31, 751–763. doi: 10.1080/01490451.2013.822615

[ref73] Vos P. Garrity G. Jones D. Krieg N. R. Ludwig W. Rainey F. A.. (2011). Bergey's manual of systematic bacteriology. United States: Springer Science & Business Media.

[ref74] Wakelin S. A. Warren R. A. Harvey P. R. Ryder M. H. (2004). Phosphate solubilization by *Penicillium* spp. closely associated with wheat roots. Biol. Fertil. Soils 40, 36–43. doi: 10.1007/s00374-004-0750-6

[ref75] Wang X. Jia Z. Liang L. Yang B. Ding R. Nie J.. (2016). Impacts of manure application on soil environment, rainfall use efficiency and crop biomass under dryland farming. Sci. Rep. 6:20994. doi: 10.1038/srep20994, PMID: 26869520 PMC4751486

[ref76] Wei Y. Zhao Y. Shi M. Cao Z. Lu Q. Yang T.. (2018). Effect of organic acids production and bacterial community on the possible mechanism of phosphorus solubilization during composting with enriched phosphate-solubilizing bacteria inoculation. Bioresour. Technol. 247, 190–199. doi: 10.1016/j.biortech.2017.09.092, PMID: 28950126

[ref77] William S. Feil H. Copeland A. (2012). Bacterial genomic DNA isolation using CTAB. Sigma 50, 1–4.

[ref78] Xiao C. Wu X. Zhu L. Yu T. Xu Z. Chi R. (2019). Enhanced biosolubilization of mid-low grade phosphate rock by formation of microbial consortium biofilm from activated sludge. Physicochem. Problems of Mineral Process. 55, 217–224. doi: 10.5277/ppmp18123

[ref79] Yahya M. Islam E. U. Rasul M. Farooq I. Mahreen N. Tawab A.. (2021). Differential root exudation and architecture for improved growth of wheat mediated by phosphate solubilizing Bacteria. Front. Microbiol. 12:744094. doi: 10.3389/fmicb.2021.744094 34721342 PMC8554232

[ref9002] Yasmin S. Zaka A. Imran A. Zahid M. A. Yousaf S. Rasul G.. (2016). Plant growth promotion and suppression of bacterial leaf blight in rice by inoculated bacteria. PloS one, 11:e0160688. doi: 10.1371/journal.pone.0160688 27532545 PMC4988697

[ref81] Zhu F. Qu L. Hong X. Sun X. (2011). Isolation and characterization of a phosphate-solubilizing halophilic bacterium Kushneria sp. YCWA18 from Daqiao Saltern on the coast of Yellow Sea of China. Evid. Based Complement. Alternat. Med. 2011, 1–6. doi: 10.1155/2011/615032, PMID: 21716683 PMC3118493

