# Supplementary material for: Development of bacteria-based bioorganic phosphate fertilizer enriched with rock phosphate for sustainable wheat production
Source: Front Microbiol. 2024 Jul 23;15:1361574. doi: 10.3389/fmicb.2024.1361574 (PMC11300357; doi:10.3389/fmicb.2024.1361574)
Supplement: Supplementary file 1 [file Table_1.DOCX]

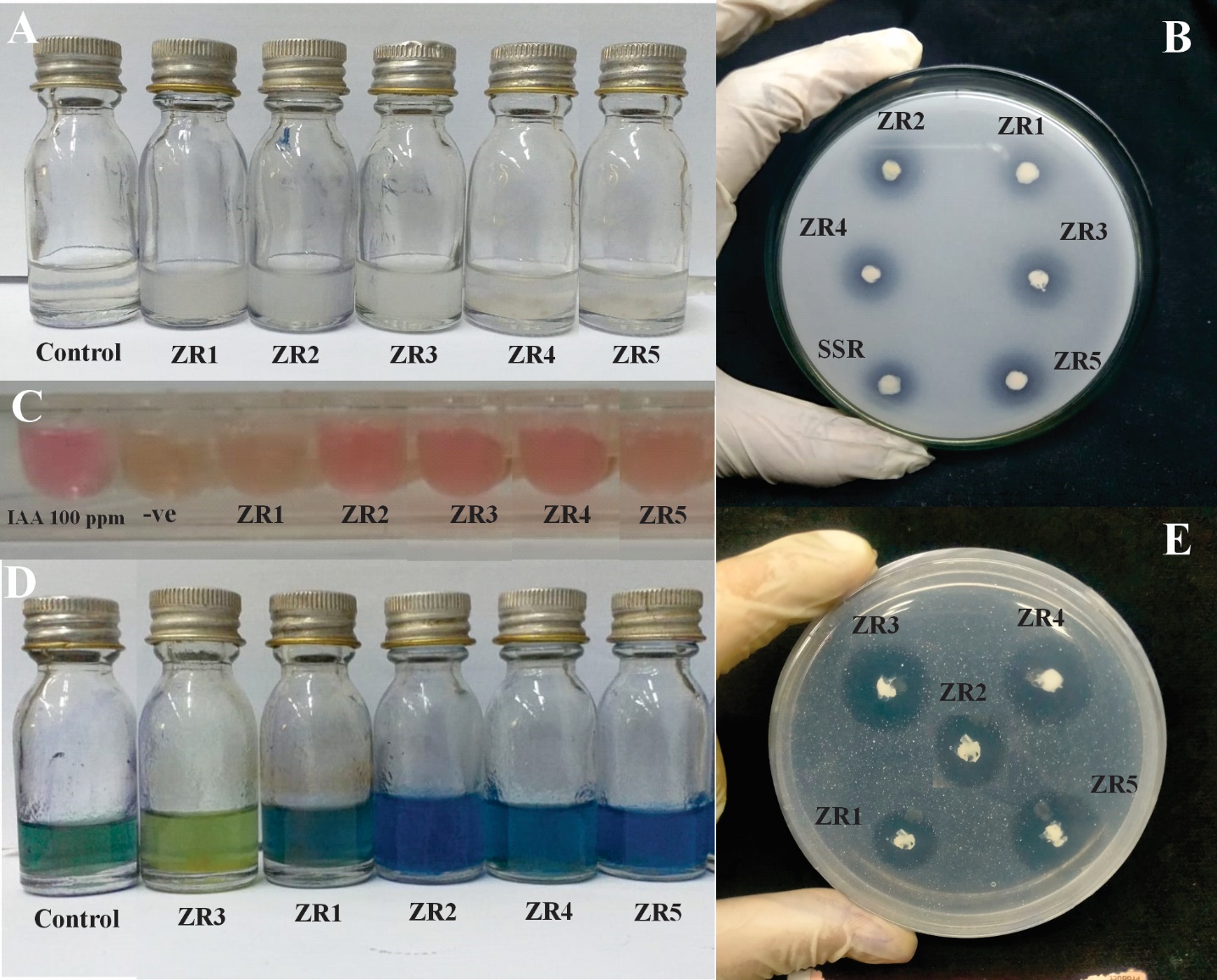


**Figure S1: Plant Growth Promoting Attributes of PSB**

A: ACC deaminase activity B: P solubilization C: IAA production D: Growth in NFM vials E: Zinc solubilization


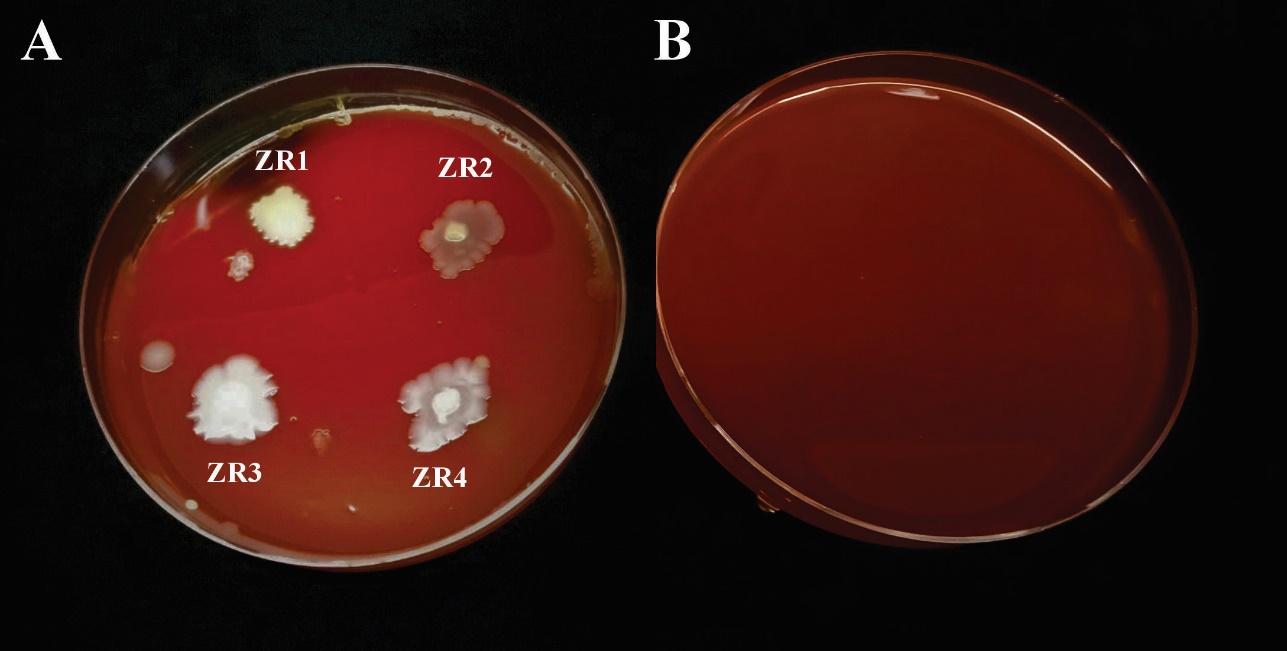


**Figure S2: Blood Agar test for Biosafety assessment**


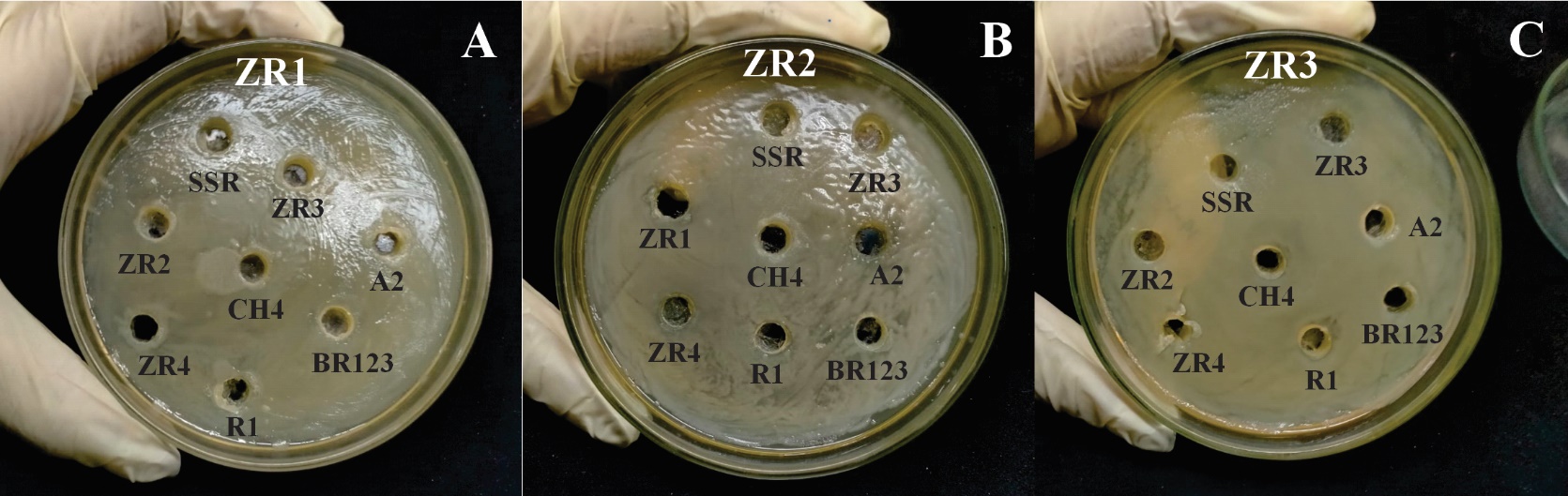


**Figure S3: Compatibility test of PSB for Consortium Development**


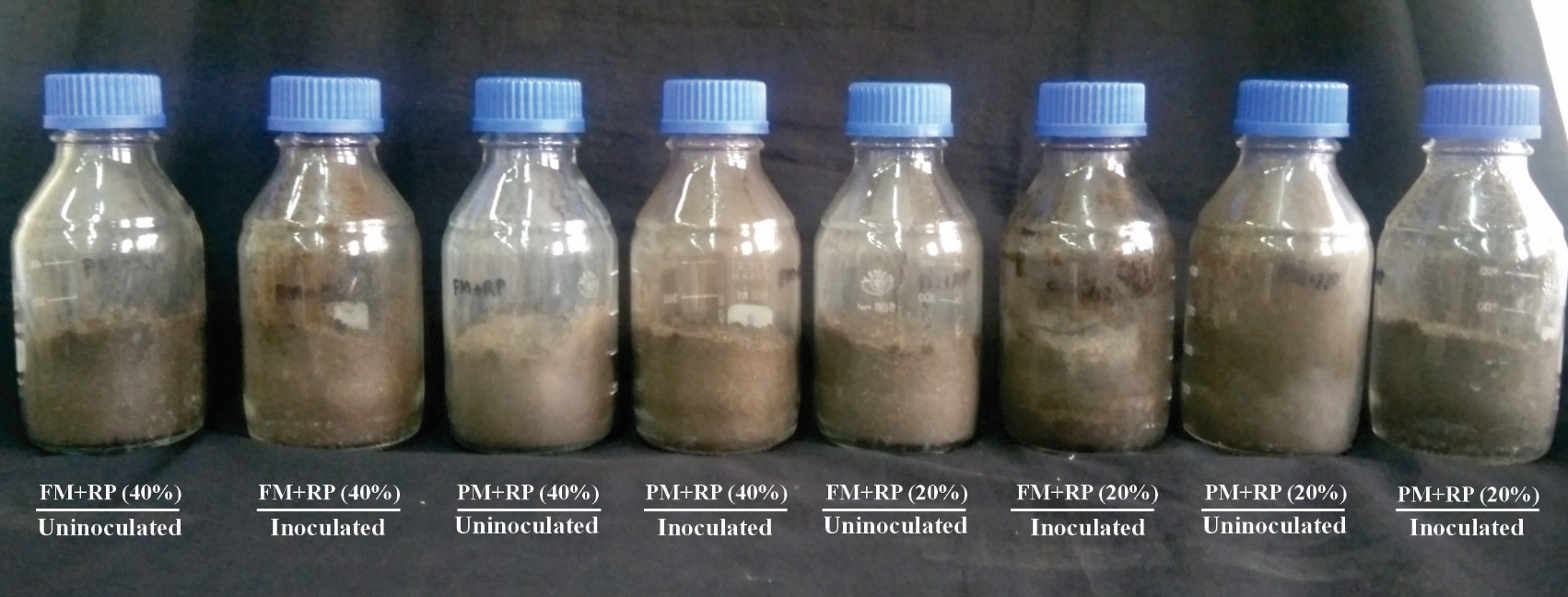


**Figure S4: Setup of microcosm bottles**
