# Supplementary material for: Development of bacteria-based bioorganic phosphate fertilizer enriched with rock phosphate for sustainable wheat production
Source: Front Microbiol. 2024 Jul 23;15:1361574. doi: 10.3389/fmicb.2024.1361574 (PMC11300357; doi:10.3389/fmicb.2024.1361574)
Supplement: Supplementary file 2 [file Table_2.DOCX]

**Table S1: Physiochemical properties of wheat rhizospheric soils collected for bacterial isolations from different sites of South Punjab, Pakistan.**

| **Sample no.** | **District** | **Coordinates** | **pH** | **EC**  **(dS m^-1^)** | **Organic**  **Matter (%)** | **Available P (mg kg^-1^)** | **Exch. K**  **(mg kg^-1^)** |
| --- | --- | --- | --- | --- | --- | --- | --- |
| **1** | Islamabad (Site 1) | 33.591068, 73.321415 | 7.99 | 1.49 | 0.71 | 1.00 | 390 |
| **2** | Bagh | 34.029349, 73.754826 | 7.73 | 0.95 | 0.65 | 17.40 | 454 |
| **3** | Islamabad (Site 2) | 33.566515, 73.272147 | 8.02 | 2.00 | 0.58 | 9.50 | 400 |
| **4** | Rawalpindi | 33.608787, 73.429849 | 7.87 | 0.99 | 0.68 | 13.85 | 218 |
| **5** | Bahawalnagar (Site 1) | 29.348194, 72.995185 | 7.84 | 0.25 | 0.41 | 6.25 | 154 |
| **6** | Multan (Site 1) | 30.268453, 71.415518 | 7.91 | 0.28 | 0.38 | 12.95 | 184 |
| **7** | Muzzafargarh | 30.282401, 71.391901 | 8.47 | 0.32 | 0.38 | 18.20 | 280 |
| **8** | Multan (Site 2) | 30.246454, 71.480404 | 7.83 | 0.31 | 0.15 | 5.95 | 134 |
| **9** | Bahawalnagar (Site 2) | 29.321651, 72.875404 | 7.92 | 0.34 | 0.59 | 20.45 | 176 |
| **10** | Sialkot | 32.429799, 74.609782 | 7.62 | 0.71 | 0.38 | 16.65 | 212 |
| **11** | Jhang (Site 1) | 31.320934, 72.599428 | 7.80 | 0.42 | 0.35 | 4.45 | 212 |
| **12** | Jhang (Site 2) | 31.288285, 72.366462 | 7.93 | 0.23 | 0.50 | 32.10 | 180 |
| **13** | Mankera (Site 1) | 31.346443, 71.682785 | 8.20 | 0.12 | 0.18 | 7.45 | 116 |
| **14** | Mankera (Site 2) | 31.276066, 71.714639 | 8.00 | 0.13 | 0.48 | 9.60 | 136 |

**Table S2: Morpho-physiological characterization of P solubilizing bacteria**

|  |  |  | **Colony Morphology** | | **Cell Morphology** | | | |
| --- | --- | --- | --- | --- | --- | --- | --- | --- |
| **Strains** | **Phosphate Solubilizing Bacteria** | **Accession numbers** | **Colony Characteristics** | **Picture** | **Shape** | **Motility** | **Gram Staining** | **Cell Picture** |
| **ZR1** | *Pantoea cypripedii* | OR272290 | Circular, Pale white, Raised, Smooth | 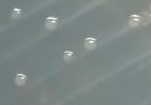 | Rods | + | - | 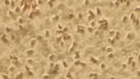 |
| **ZR2** | *Lysinibacillus fusiformis* | OR272301 | Circular, White, Raised, Smooth and Shiny | 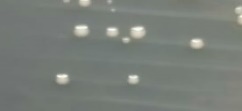 | Rods | - | - | 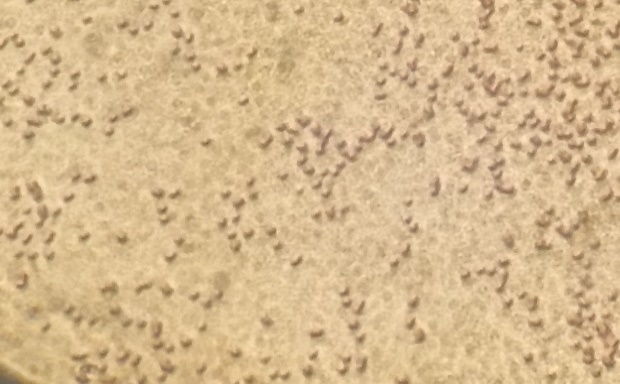 |
| **ZR3** | *Enterobacter quasihormaechei* | OR272303 | Off-White, Flat, Irregular-edged colonies. | 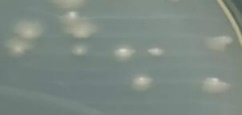 | Rods | ++ | - | 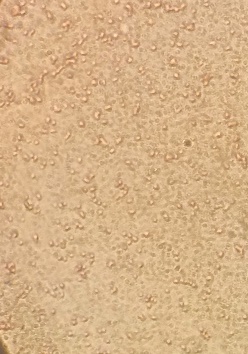 |
| **ZR4** | *Klebsiella pneumoniae* | OR272305 | Circular, Creamy White, Convex, Raised, Smooth, Shiny, Sticky colonies | 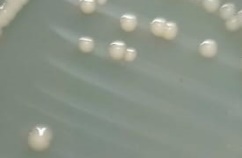 | Small Rods | - | - | 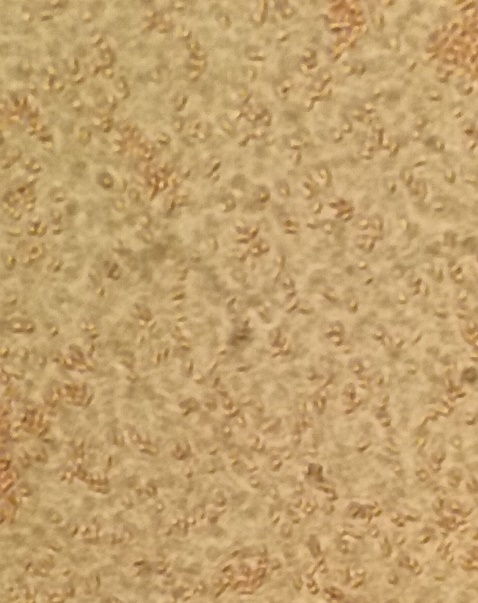 |
| **ZR5** | *Klebsiella quasivariicola* | OR272306 | Circular, White, Entire, Flat, Smooth, Sticky colonies | 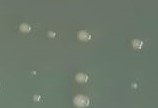 | Small Rods | - | - | 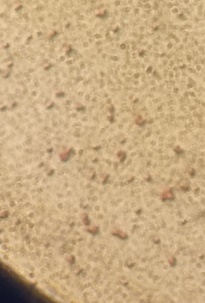 |
